# Global Diversity and Biogeography of the *Zostera marina* Mycobiome

**DOI:** 10.1128/AEM.02795-20

**Published:** 2021-05-26

**Authors:** Cassandra L. Ettinger, Laura E. Vann, Jonathan A. Eisen

**Affiliations:** a Genome Center, University of California, Davis, Davis, California, USA; b Department of Evolution and Ecology, University of California, Davis, Davis, California, USA; c Department of Genomics and Bioinformatics, Novozymes, Davis, California, USA; d Department of Medical Microbiology and Immunology, University of California, Davis, Davis, California, USA; Wageningen University

**Keywords:** seagrasses, *Zostera marina*, marine fungi, microbial eukaryotes, 18S rRNA, ITS2, eelgrass, mycobiome, core, abundance-occupancy, dispersal limited, plant selected, global distribution

## Abstract

Seagrasses are marine flowering plants that provide critical ecosystem services in coastal environments worldwide. Marine fungi are often overlooked in microbiome and seagrass studies, despite terrestrial fungi having critical functional roles as decomposers, pathogens, or endophytes in global ecosystems. Here, we characterize the distribution of fungi associated with the seagrass *Zostera marina,* using leaves, roots, and rhizosphere sediment from 16 locations across its full biogeographic range. Using high-throughput sequencing of the ribosomal internal transcribed spacer (ITS) region and 18S rRNA gene, we first measured fungal community composition and diversity. We then tested hypotheses of neutral community assembly theory and the degree to which deviations suggested that amplicon sequence variants (ASVs) were plant selected or dispersal limited. Finally, we identified a core mycobiome and investigated the global distribution of differentially abundant ASVs. We found that the fungal community is significantly different between sites and that the leaf mycobiome follows a weak but significant pattern of distance decay in the Pacific Ocean. Generally, there was evidence for both deterministic and stochastic factors contributing to community assembly of the mycobiome, with most taxa assembling through stochastic processes. The *Z. marina* core leaf and root mycobiomes were dominated by unclassified Sordariomycetes spp., unclassified Chytridiomycota lineages (including Lobulomycetaceae spp.), unclassified Capnodiales spp., and *Saccharomyces* sp. It is clear from the many unclassified fungal ASVs and fungal functional guilds that knowledge of marine fungi is still rudimentary. Further studies characterizing seagrass-associated fungi are needed to understand the roles of these microorganisms generally and when associated with seagrasses.

**IMPORTANCE** Fungi have important functional roles when associated with land plants, yet very little is known about the roles of fungi associated with marine plants, like seagrasses. In this study, we report the results of a global effort to characterize the fungi associated with the seagrass *Zostera marina* across its full biogeographic range. Although we defined a putative global core fungal community, it is apparent from the many fungal sequences and predicted functional guilds that had no matches to existing databases that general knowledge of seagrass-associated fungi and marine fungi is lacking. This work serves as an important foundational step toward future work investigating the functional ramifications of fungi in the marine ecosystem.

## INTRODUCTION

Terrestrial fungi are known to have critical ecological roles as microbial saprotrophs, pathogens, and mutualists (1), and although less is known about fungi in aquatic ecosystems, it is believed that they also have vital ecological roles (e.g., in organic matter degradation, nutrient cycling, and food web dynamics [2–7]). Despite their global importance, the taxonomic, phylogenetic, and functional diversity of marine fungi generally is vastly understudied (8). In comparison to the greater than 120,000 terrestrial fungal species known (9), there are currently only ∼1,900 described species of marine fungi, though estimates of the true diversity of these organisms are much higher (10–12). Recent studies have examined the global distribution of marine planktonic, pelagic, and benthic fungi (13–15), yet the distribution of host-associated fungi in the marine environment is still relatively unknown. Fungi have been reported in association with many marine animals, including sponges (16), corals (17), and other invertebrates (18), with algae and seaweeds (19, 20), and with flowering plants, like seagrasses (21).

Seagrasses are foundation species in coastal ecosystems worldwide and are the only submerged angiosperms (flowering plants) to inhabit the marine environment. One widespread seagrass species, Zostera marina, also known as eelgrass, provides critical ecosystem services in coastal environments throughout much of the Northern Hemisphere (22–24). Previous studies have investigated the composition and structure of the bacterial community associated with *Z*. *marina*, including a global survey that was able to identify a core eelgrass root microbiome (25–27). Members of this community are thought to facilitate nitrogen and sulfur cycling for host plant benefit (25, 27–33).

Comparatively, not as much is known about the distribution, diversity, and function of the mycobiome (i.e., the fungal community) associated with *Z*. *marina*. Culture-based studies have described a mycobiome composed of taxa in the classes Eurotiomycetes, Dothideomycetes, and Sordariomycetes (34–37). These studies consistently find dominance of a few ubiquitous taxa (e.g., *Cladosporium* sp.) but also a diverse set of rare taxa that vary among sites and may be endemic to specific locations (e.g., *Colletotrichum* sp.) (37). One hypothesis for this pattern is that the fungal community is assembled neutrally through stochastic processes.

While culture-independent studies of *Z. marina* and other seagrass species have more exhaustively characterized the taxonomic diversity of these fungal communities, they have also highlighted how little is known about factors affecting the distribution, function, and community assembly of seagrass-associated fungi (38–42). A common finding among these studies is that taxonomic assignments cannot be made for greater than two-thirds of the fungal sequences associated with seagrasses and that Chytridiomycota lineages are dominant in this ecosystem (39, 40, 42). Culture-independent understanding of the mycobiome of *Z. marina* has so far focused on a single location in Bodega Bay, California (42). However, site-to-site variation in the mycobiome has now been observed in mycobiome studies from several other seagrass species (38–41). For example, a distance decay relationship was found for the fungal community associated with the seagrass Enhalus acoroides in Singapore and Peninsular Malaysia (40) and for the seagrass Syringodium isoetifolium along Wallace’s Line, a boundary line separating Asian and Australasian taxa (41). Additionally, the global planktonic marine fungal community was found to cluster by ocean (15); therefore, we hypothesize in this study that in addition to a distance decay relationship, we will observe differentiation by ocean basin. Such geographic relationships would support an alternative hypothesis of niche-based community assembly of the mycobiome through deterministic processes, such as environmental filtering and dispersal.

One concept central to the investigation here is the role of stochastic and deterministic drivers in determining the community assembly of the seagrass mycobiome. The Sloan neutral model assumes that random immigrations, births, and deaths determine the relative abundance of taxa in a community (43, 44). The model further assumes that local communities are assembled stochastically from regional pools and that deterministic competitive interactions are not important because species are competitively equivalent (45–47). Stochastic processes supporting the neutral model include priority effects and ecological drift, while deterministic processes include species traits, interspecies interactions (e.g., competition, mutualisms), and environmental conditions (48). Dispersal limitation can be either a stochastic or deterministic process (49). Identifying specific taxa that deviate from the model allows us to predict taxa that are assembled through deterministic processes, including plant selection (50).

Here, we use high-throughput sequencing of marker genes to (i) characterize the fungal community associated with the seagrass *Zostera marina* on a global scale, (ii) test for a pattern of distance decay within ocean basins, (iii) define a global core mycobiome, (iv) use neutral models to test whether the community is assembled through a stochastic, deterministic, or combination of processes, (v) predict important fungal taxa based on assembly dynamics and global distribution, and (vi) assign functional predictions for the fungal community associated with *Z. marina*.

## RESULTS

### Fungal alpha diversity differs between sites, tissues, and oceans.

**FIG 1:**
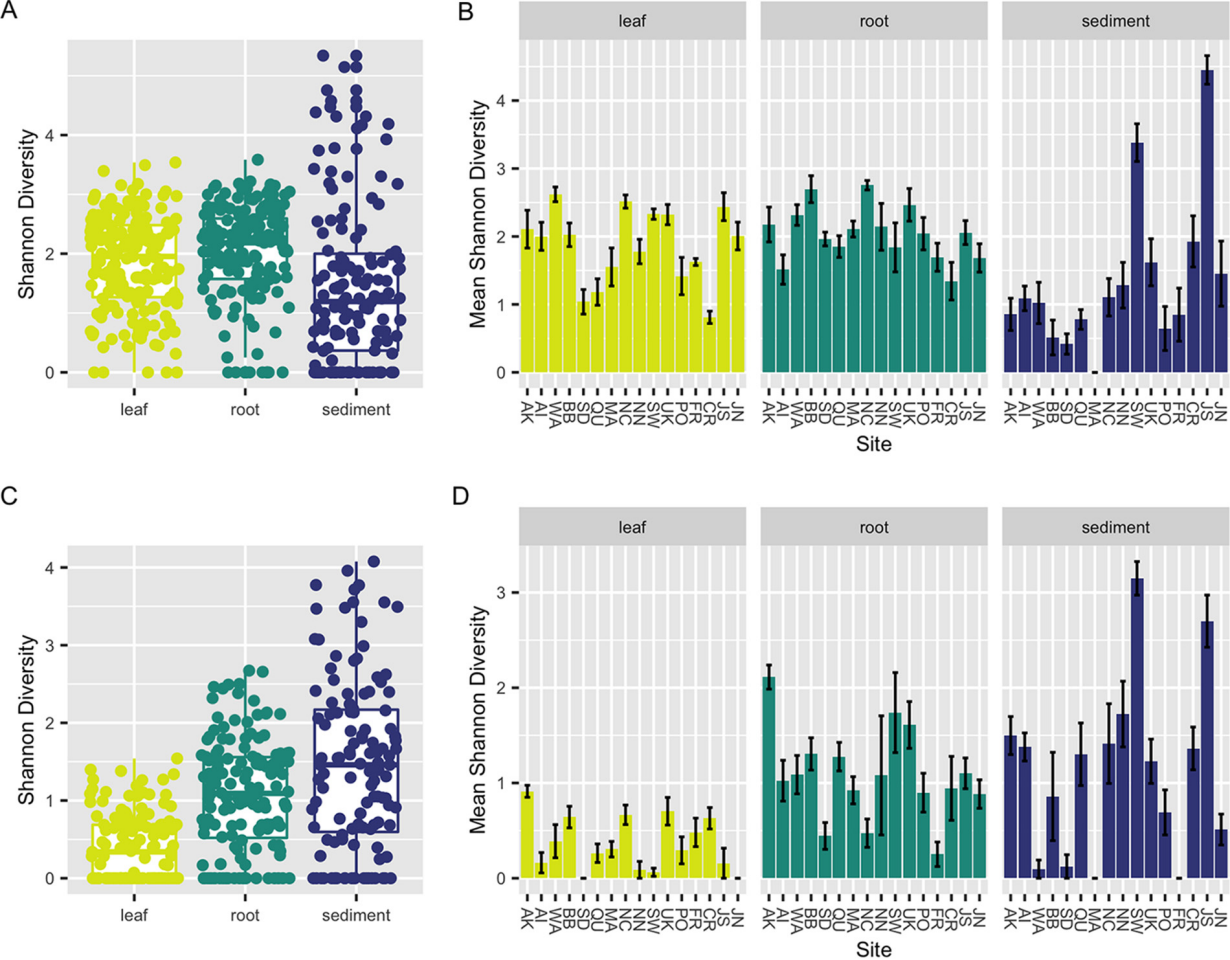
Within-sample diversity varies across tissues and sites. Here we depict sample Shannon diversities for the ITS2 region for each sample type (leaf, root, sediment) using box plots (A) and within each sample type at each collection site (see Table 1 for site codes) using bar charts (B). Shannon diversities for the 18S rRNA gene amplicon data are also plotted for each sample type (leaf, root, sediment) using box plots (C) and within each sample type at each collection site (see Table 1 for site codes) using bar charts (D). For panels B and D, the standard error of the mean Shannon diversity at each site for each sample type is represented by an error bar, and bars are colored by sample type. *Post hoc* Dunn results for pairwise site comparisons for each sample type can be found in Tables S1 to S6 in the supplemental material. For panels A to D, zero values indicate samples that are dominated by a single taxon.

The Shannon index was significantly different between sample types (Kruskal-Wallis [K-W] test, *P < *0.001) (Fig. 1) for both the internal transcribed spacer 2 (ITS2) region amplicon and 18S rRNA gene amplicon data sets. *Post hoc* Dunn tests of both data sets suggest that alpha diversity for leaves was generally lower than that for roots (*P < *0.05). However, there were conflicting results for the sediment, with diversity being lower in the sediment than in leaves and roots in the ITS2 region amplicons (*P < *0.05) and diversity being higher in the sediment than in leaves and roots in the 18S rRNA gene amplicons (*P < *0.05). Alpha diversity for both data sets also was significantly different within each sample type across sites (K-W test, *P < *0.001) (Fig. 1). This was driven by diversity being significantly different across some but not all sites (Dunn test, *P < *0.05) (see Tables S1 to S6 in the supplemental material). For leaves, this was caused by lower diversity at some sites (SD, CR, QU [see Table 1 for site codes]) in the ITS2 region amplicon data and higher diversity of leaves at one site (AK) in the 18S rRNA gene amplicon data. For roots, this was driven by higher diversity at two sites (NC, BB) in the ITS2 region amplicon data and, for the 18S rRNA gene data, higher diversity at one site (AK) and lower diversity at others (SD, NC, FR). For the sediment for both data sets, observed alpha diversity differences were due to higher diversity at two sites (JS, SW). Alpha diversity for leaves was significantly different across oceans for the ITS2 region amplicon data set (K-W test, *P = *0.014) but was not significantly different for roots or sediment between oceans or for leaves, roots, or sediment between oceans for the 18S rRNA gene amplicon data set (*P > *0.05).

**TABLE 1:** Description of collection sites^*a*^

Site code	Site	Site name	Collection mo	Collection yr	Latitude	Longitude
AK	Alaska—North	Kotzebue	October	2016	64.485428	−164.76189
AI	Alaska—South	Izembek	October	2016	55.328899	−162.82121
BB	California—North	Westside Park, Bodega Bay	December	2016	38.319755	−123.05514
SD	California—South	Shelter Island	November	2016	32.713756	−117.22547
QU	Canada	Pointe-Lebel	September	2016	49.11237	−68.17593
CR	Croatia	Posedarje	September	2016	44.21155	15.4906946
FR	French Mediterranean	Bouzigues, Etang de Thau	October	2016	43.446971	3.661503
JN	Japan—North	Akkeshi-ko estuary	September	2016	43.021167	144.903217
JS	Japan—South	Ikunoshima	September	2016	34.297834	132.91631
MA	Massachusetts	Dorothy Cove	October	2016	42.42014	−70.91544
NC	North Carolina	Middle Marsh	April	2017	34.692458	−76.622589
NN	Norway	Røvika	July	2016	67.2667233	15.2560633
PO	Portugal	Culatra	October	2016	37.01427	−7.493273
SW	Sweden	Torseröd	August	2016	58.3131	11.5488
UK	Wales	Porthdinllaen	March	2017	52.990731	−4.450321
WA	Washington	Willapa Bay	September	2016	46.474	−124.028

a Specifics of each collection site, including the site code, site name, month of sample collection, year of sample collection, and site latitude and longitude, are given.

### Fungal community structure differs across sites, tissues, and oceans.

**FIG 2:**
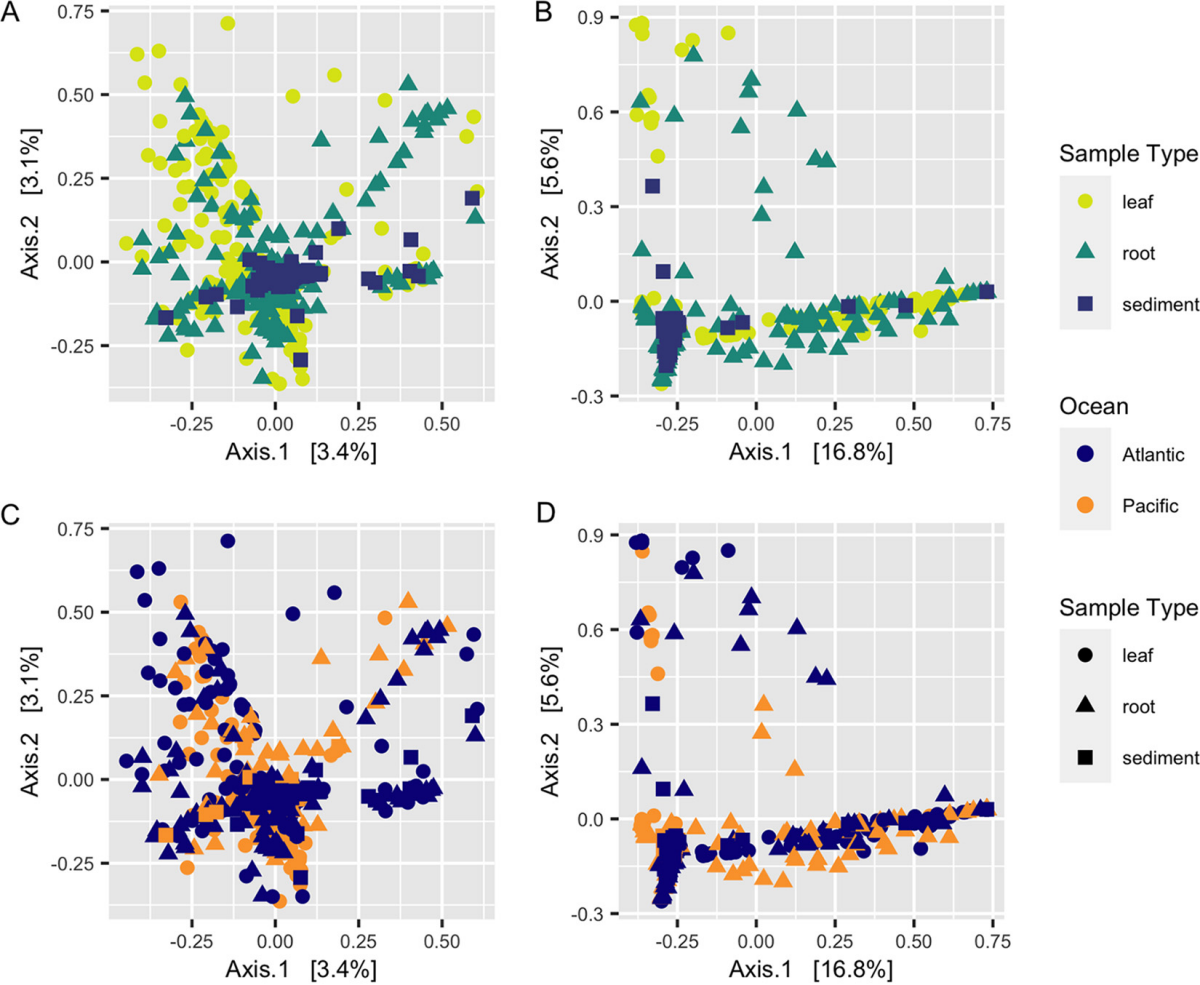
Community structure varies between tissues and ocean basins. Principal-coordinate analysis (PCoA) visualization of Hellinger distances of fungal communities associated with leaves, roots, and sediment based on ITS2 region amplicon data (A and C) and 18S rRNA gene amplicon data (B and D). (A and B) Points in the ordination are colored and represented by shapes based on sample type: leaf, yellow circles; root, green triangles; sediment, blue squares. (C and D) Points in the ordination are colored by ocean (Pacific, blue; Atlantic, orange) and represented by shapes based on sample type (circles), root (triangles), or sediment (squares).

Similar to alpha diversity, fungal beta diversity was significantly different for both data sets using all three ecological metrics (Bray-Curtis dissimilarity, Aitchinson distance, Hellinger distance) across sample types (permutational multivariate analysis of variance [PERMANOVA], *P < *0.001) (Fig. 2A and B), across sites (*P < *0.001) (Fig. S1), and across oceans (*P < *0.001) (Fig. 2C and D). *Post hoc* pairwise PERMANOVA tests using the ITS2 region amplicon data indicated significant differences in beta diversity across all sample types (*P < *0.001) and all sites (*P < *0.01). These results were generally consistent with the 18S rRNA gene sequence data, which supported differences in community structure across sample types (*P < *0.001) and across the majority of, but not all, collection sites (*P < *0.05).

Within-group variance (i.e., dispersion) also differed significantly for the ITS2 region amplicon data using all three beta diversity metrics across sample types (betadisper, *P < *0.01) and sites (*P < *0.01) but did not vary across oceans (*P > *0.05). Mean dispersion between sites in the 18S rRNA gene data was not significant for two of the ecological metrics (Bray-Curtis, *P = *0.79; Hellinger, *P = *1), and similarly, the mean dispersion between oceans was not significant for two of the ecological metrics (Bray-Curtis, *P = *0.07; Aitchinson, *P = *0.26). Mean dispersion was otherwise consistent with the significant results observed in the ITS2 region amplicon data. PERMANOVA results have been shown to confuse dispersion differences and centroid differences when a balanced design is not used. Therefore, these results may indicate that either mean centroids, mean dispersions, or both are differing between sample types and sites here.

### Mantel tests suggest weak distance decay relationships within oceans.

**FIG 3:**
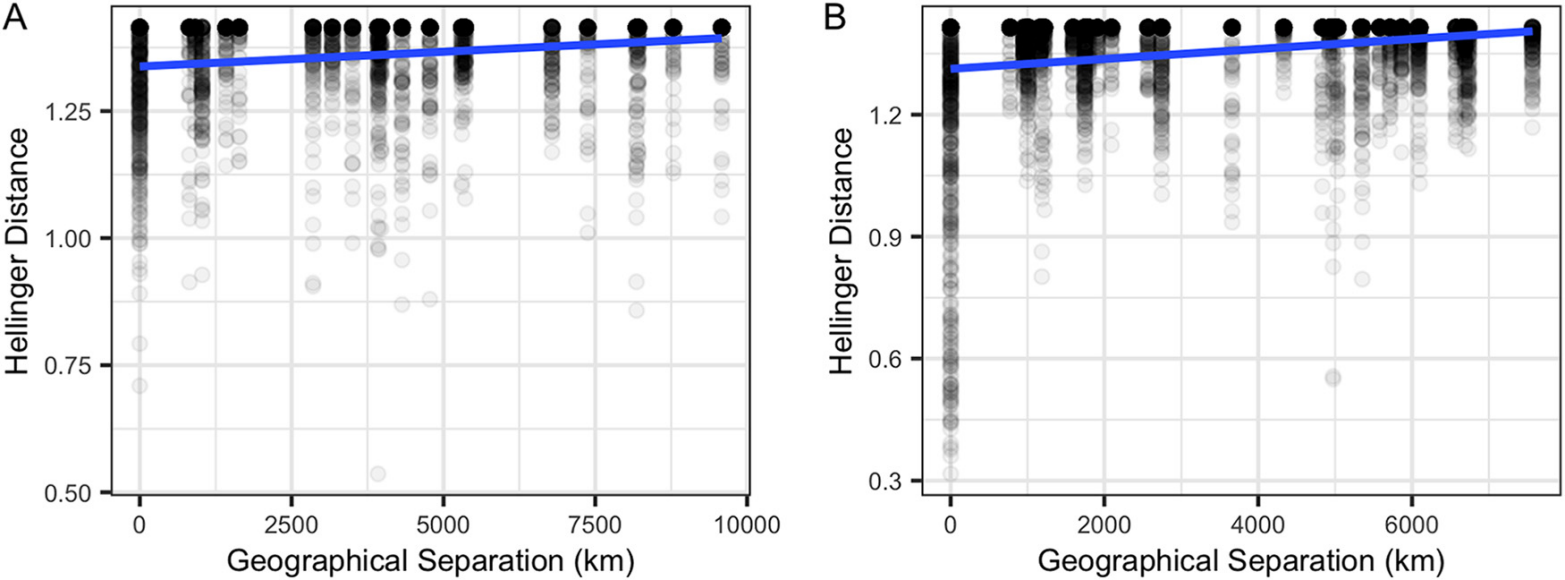
Mantel tests suggest a distance decay relationship. Scatterplots depict the weak but significant positive distance-decay relationship between leaf fungal community beta diversity (Hellinger distance) using the ITS2 region amplicon data and geographical distance (km) between sites from the Pacific Ocean (*r *= 0.1767, *P = *0.0001) (A) and Atlantic Ocean (*r *= 0.1057, *P = *0.0001) (B).

Mantel tests indicated a small, but significant positive relationship between both metrics of community structure (Bray-Curtis, Hellinger) and geographic distance for leaves across the Pacific Ocean for the ITS2 region and 18S rRNA gene amplicon data sets (*P < *0.001) (Fig. 3A; Fig. S2A and Table S7). This relationship was also detected for leaves across the Atlantic Ocean (*P < *0.001) (Fig. 3B; Fig. S2B and Table S7). Mantel correlograms suggest that this pattern is driven by sites with the closest proximity, such that sites closer together have more similar fungal communities than sites further away (Fig. S3). This pattern is sustained for both data sets for the Pacific Ocean but not for the Atlantic Ocean, when community structure comparisons from the same location are removed (*P < *0.05) (Table S7). In the Pacific Ocean, roots had a positive relationship with geographic distance for both the ITS2 region and 18S rRNA gene amplicon data sets (*P < *0.001) (Fig. S2C and S4A and Table S7), and an even stronger significant positive relationship was observed for roots in the Atlantic Ocean (*P < *0.001) (Fig. S2D and S4B and Table S7). However, upon removal of comparisons from the same location, a pattern of distance decay was observed only in the 18S rRNA gene data set for roots in the Atlantic Ocean (*P < *0.001) (Table S7), suggesting that the earlier observed pattern in the Pacific was driven by high similarity in community structure between roots at the same site. Sediment in the Pacific Ocean had a weak relationship with distance, with conflicting significance for the ITS2 region (Bray-Curtis, *P < *0.001; Hellinger, *P = *0.824), but had strong significant correlations for 18S rRNA gene amplicon data sets (*P < *0.001) (Fig. S2E and S4C and Table S7). Upon removal of beta diversity comparisons from the same location, no distance decay pattern was observed in the Pacific (*P > *0.05) (Table S7). In contrast, in the Atlantic Ocean, sediment had much more robust positive relationships with geographic distance for both data sets (*P < *0.001) (Fig. S2F and S4D and Table S7), which generally remained even when comparisons from the same location were excluded (Table S7). Multiple-regression analyses further confirmed all significant patterns of observed distance decay (*P < *0.001).

### Global core leaf, root, and sediment mycobiomes.

**FIG 4:**
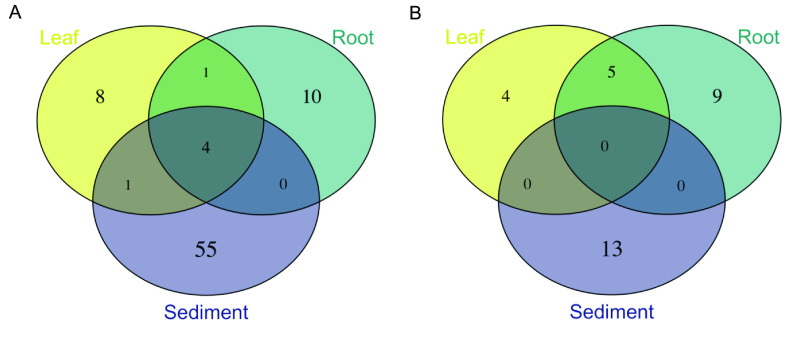
Overlap between predicted core mycobiomes of individual *Z. marina* tissues. Venn diagrams representing shared core ASVs as defined by abundance-occupancy distributions for each sample type (leaf, root, sediment) for ITS2 region amplicon data (A) and 18S rRNA gene amplicon data (B). For comparison, overlap of the entire mycobiome of individual *Z. marina* tissues is shown in Fig. S5 in the supplemental material.

We utilized abundance-occupancy distributions of amplicon sequence variants (ASVs) to infer global *Z. marina* leaf, root, and rhizosphere sediment core mycobiomes based on ASV rank contributions to beta diversity. A total of 14, 15, and 60 ASVs were predicted as being in the leaf, root, and sediment cores, respectively, based on the ITS2 region amplicon data (Fig. 4A; Table S8). Four ASVs overlapped across all three cores; this included generalist fungi with widespread distributions such as *Cladosporium* sp. and Malassezia restricta (37, 51). Interestingly, only one ASV was shared between leaf and root cores, Saccharomyces paradoxus (ITS_SV260). The leaf core was dominated by unclassified Capnodiales spp., while the root core was dominated by unclassified Sordariomycetes spp. The sediment core was more diverse but was composed mostly of Ascomycota, particularly members in the Pleosporales and Agaricales.

Smaller core mycobiomes were predicted from the 18S rRNA gene amplicon data with only 9, 14, and 13 ASVs placed in the leaf, root, and sediment cores, and no ASVs overlapped between the three cores (Fig. 4B; Table S9). Generally unclassified Chytridiomycota lineages (including Lobulomycetaceae spp.), Sordariomycetes spp., and Saccharomycetales sp. dominated these core communities.

### Neutral models to predict ASV selection.

**FIG 5:**
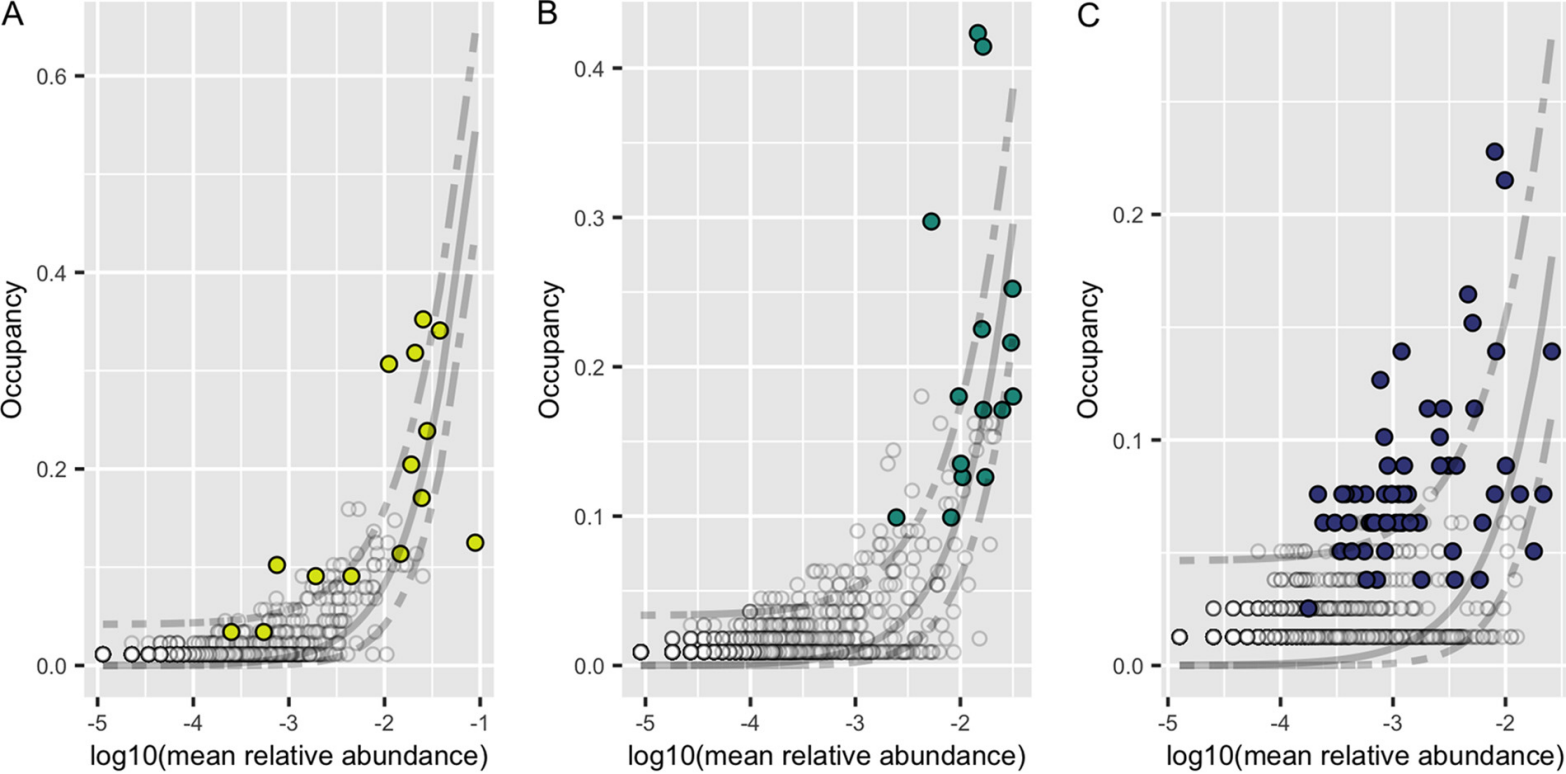
Abundance-occupancy distributions reveal core mycobiomes. Abundance-occupancy distributions were used to define core members of the leaf (A), root (B), and sediment (C) mycobiomes for the ITS2 region amplicon data. Each point represents an ASV, with predicted core members indicated by a color (leaf = yellow, root = green, sediment = blue) and noncore ASVs in white. Ranked ASVs were predicted to be in the core based on a final percent increase equal to or greater than 10%. A solid line represents the fit of the neutral model, and dashed lines represent 95% confidence around the model prediction. ASVs above the neutral model are predicted to be selected for by the environment (e.g., by the host plant, *Z. marina*), and those below the model are predicted to be selected against or dispersal limited.

We applied Sloan neutral models to investigate if ASVs are selected for by *Z. marina*, assembled through stochastic or deterministic processes (43, 44). ASVs that fall above the neutral model prediction appear in higher occupancy than would be predicted based on their relative abundance and are thus thought to be selected for by the plant environment. ASVs that fall below the neutral model prediction have higher relative abundance than would be predicted based on their occupancy and are thus thought to be either selected against by the plant host or dispersal limited. For the ITS2 region abundance-occupancy distributions, 2.9%, 4.8%, and 3.7% of all ASVs fell above/below the neutral model prediction for leaves, roots, and sediment, respectively (Fig. 5), while for the 18S rRNA gene abundance-occupancy distributions, 7.5%, 6.4%, and 2.2% of all ASVs deviated from the neutral model prediction for leaves, roots, and sediment, respectively (Fig. S6). Further, looking at deviations from the neutral model for ASVs predicted to be in the core mycobiome allows insight into the role of *Z. marina* in core assembly. We found that of the core leaf, core root, and core sediment ASVs, several were predicted to be plant selected (*n*_leaf_ = 6, *n*_root_ = 7, *n*_sediment_ = 40), only a few were selected against or dispersal limited (*n*_leaf_ = 1, *n*_root_ = 3, *n*_sediment_ = 4), and most were neutrally selected (*n*_leaf_ = 16, *n*_root_ = 19, *n*_sediment_ = 29).

### Considering the fit of the neutral model.

Generally, the neutral model had a poor fit for both the ITS2 region (leaf, *R^2^* = 0.31; root, *R^2^* = 0.44; sediment, *R^2^* = −0.76) and 18S rRNA gene (leaf, *R^2^* = 0.49; root, *R^2^* = 0.50; sediment, *R^2^* = 0.08) data sets, with the sediment curves having the worst fit to the neutral model. This could potentially be attributed to the low predicted migration rates (*m*) for both the ITS2 region (leaf, *m *= 0.001; root, *m *= 0.002; sediment, *m *= 0.001) and 18S rRNA gene (leaf, *m *= 0.002; root, *m *= 0.014; sediment, *m *= 0.014) data sets. These values are consistent with other studies of fungi that used neutral models on abundance-occupancy curves (52) and may be reflective of dispersal limitation playing a stronger role in fungal assembly than bacterial community assembly (53–55).

### Global distribution of differentially abundant ASVs.

To investigate variation in fungal community composition at greater taxonomic resolution, we used DESeq2 to identify ASVs whose abundance differed across sample types (Fig. S7 and S8). The greatest number of differentially abundant ASVs was observed between the roots and sediment, with 14 ITS2 region ASVs and four 18S rRNA gene ASVs (Wald test, *P < *0.01). This was closely followed by differentially abundant ASVs between leaves and sediment, with 12 ITS2 region ASVs and two 18S rRNA gene ASVs (*P < *0.05). The smallest number of differentially abundant ASVs was found between leaves and roots, with three ITS2 region ASVs (*P < *0.01). We compared the differentially abundant ASVs to those predicted to be in the leaf, root, and sediment core mycobiomes. We found 14 ASVs that were both differentially abundant between sample types and present in at least one core mycobiome; of those 14, seven were also found to deviate from the neutral model (Table 2). We then examined the global distribution of the 14 ASVs that were both differentially abundant across sample types and predicted to be in the *Z. marina* core mycobiome. For example, ITS_SV260 (Saccharomyces paradoxus) appears to be globally distributed, neutrally selected, and more abundant on leaves and roots than sediment (*P < *0.001) (Fig. S9). In contrast, ITS_SV362 (*Lobulomyces* sp.) appears to be found only at one site and dispersal limited and is more abundant on leaves than sediment (*P < *0.001) (Fig. S10).

**TABLE 2:** Predicted differentially abundant core ASVs^*a*^

ASV	Core prediction	Neutral model deviation(s)	Significant DESeq2 comparisons	Taxonomy
ITS_SV52	Leaf, root, sediment	Above, above, none	Root > sediment	*Mycosphaerella tassiana*
ITS_SV60	Root	Below	Leaf > sediment; root > sediment	Unclassified Sordariomycetes sp.
ITS_SV125	Root	None	Root > sediment	Unclassified Ascomycota sp.
ITS_SV234	Root	None	Leaf > sediment; root > sediment	Unclassified Sordariomycetes sp.
ITS_SV260	Leaf, root	None, none	Leaf > sediment; root > sediment	Saccharomyces paradoxus
ITS_SV362	Leaf	Below	Leaf > sediment; root > sediment	*Lobulomyces* sp.
ITS_SV426	Leaf, sediment	None, none	Leaf > sediment; root > sediment	*Saccharomyces* sp.
ITS_SV497	Root	Below	Leaf > sediment; root > sediment	Unclassified Sordariomycetes sp.
ITS_SV679	Sediment	Above	Root > leaf; root > leaf	*Pseudeurotium bakeri*
ITS_SV1045	Root	Above	Leaf > sediment; root > sediment	*Hortaea werneckii*
18S_SV756	Leaf, root	None, none	Root > sediment	Unclassified Chytridiomycetes sp.
18S_SV928	Leaf, root	Above, above	Leaf > sediment; root > sediment	*Saccharomyces* sp.
18S_SV968	Leaf	None	Leaf > sediment; root > sediment	Unclassified Lobulomycetaceae sp.
18S_SV1977	Root	None	Root > sediment	*Chytridium* sp.

a ASVs were ranked by abundance-occupancy distributions and then predicted to be in a core based on a final percent increase in beta diversity equal to or greater than 10%. The Sloan neutral model was then applied to the abundance-occupancy distributions to identify ASVs that deviate such that ASVs above the neutral model are predicted to be selected for by the environment (e.g., by the host plant, *Z. marina*) and those below the model are predicted to be selected against or dispersal limited. Finally, DESeq2 was used to identify ASVs that were differentially abundant between pairwise sample types (leaf, root, sediment). Here, for each predicted core ASV that was also differentially abundant for at least one pairwise comparison, we report the ASV, the core of which it was predicted to be a member (leaf, root or sediment), whether the ASV deviated from the neutral model (above, below, or none), the significant pairwise differential abundance comparisons (e.g., root > sediment means that the ASV was in significantly higher abundance when associated with roots than with sediment), and the taxonomy of the ASV.

### Fungi are only a small portion of the *Z. marina*-associated eukaryotic community.

Fungal sequences made up only a tiny portion of the entire epiphytic eukaryotic community associated with *Z. marina*, with a mean relative abundance on leaves of 0.50% ± 2.1%, on roots of 0.12% ± 0.4%, and on sediment of 0.23% ± 0.7% in the 18S rRNA gene data set (Fig. S11). The leaf eukaryotic community was generally dominated by diatoms, the root community by both diatoms and Peronosporomycetes (i.e., oomycetes), and the sediment community by both diatoms and dinoflagellates.

### Many ASVs have no predicted functional guild.

Although FUNGuild was able to predict the functional guild and trophic mode of 78.6% of ASVs in the ITS2 region amplicon data set, only 10.1% of ASVs had predictions at a confidence of “highly probable.” The most abundant functional guilds assigned at this confidence level included wood saprotroph, ectomycorrhizal, lichenized, endophyte, plant pathogen-wood saprotroph, and fungal parasite (Fig. S12). Comparatively, FUNGuild was able to predict functions for only 35.3% of the ASVs in the 18S rRNA gene data set and only 3.4% of those ASVs had “highly probable” predictions. Generally, the most abundant functional guilds at this confidence level were consistent with those in the ITS2 region data set and included wood saprotroph, ectomycorrhizal, and plant pathogen (Fig. S13). When we further investigated the predicted trophic modes of only the most abundant ASVs (mean relative abundance greater than 0.1%) in both the ITS2 region amplicon and 18S rRNA amplicon data sets, 38.4% and 63.5% of these ASVs, respectively, were unable to be assigned a function, a testament to how little we know about the functional roles of fungi in this system.

## DISCUSSION

### Overview.

This study is the first to characterize the *Zostera marina* mycobiome across its full biogeographic distribution using culture-independent methods. We found that the fungal community was different between sites globally and observed a small but significant pattern of distance decay for the *Z. marina* mycobiome in the Pacific Ocean. We defined a small core mycobiome for leaves, roots, and sediment dominated by Sordariomycetes spp., Chytridiomycota lineages (including Lobulomycetaceae spp.), and Capnodiales spp. Finally, we found the assembly of the mycobiome of *Z. marina* to be dominated by stochastic processes, although we also observed taxa, including predicted members of the core mycobiome, whose assembly was predicted to be affected by deterministic factors (e.g., environmental filtering, host genetics, dispersal limitation).

### Fungal diversity and community structure are generally consistent with previous work.

We observed significant differences in alpha diversity across seagrass tissues, which is consistent with previous seagrass work (38, 40, 42). We also observed differences in alpha diversity across sites, which is consistent with some previous seagrass work (38, 40), while other work found no differences in alpha diversity between sites (39). Additionally, we also observed differences in fungal community structure across tissues and sites. Differences in fungal beta diversity across sites and tissues have been reported previously for seagrasses (26, 38–42). Seasonal differences in fungal colonization of seagrasses have been observed and are likely contributing to the variation observed here between sites, as we were not able to collect from all sites during the same season (56). The global planktonic marine fungal community has been found to cluster by ocean (15), and differences between oceans were observed here as well, suggesting that fungal dispersal is occurring within ocean basins. However, site-to-site variation was a stronger factor than ocean basin in explaining community structure, suggesting that local environmental or host plant filtering may play a critical role in assembling the fungal community associated with *Z. marina*.

### Possible factors driving the distance decay pattern within ocean basins.

It has long been thought that there are few barriers to fungal dispersal (57–60). However, not every fungus is everywhere (61), and there is increasing evidence for rampant environmental filtering and dispersal barriers for host-associated fungi in the marine ecosystem (40, 41, 62). The importance of biogeography for seagrass-associated fungal community structure can be seen in the observation of a small but significant positive distance decay relationship between geographic distance and leaf community structure in the Pacific Ocean and sediment in the Atlantic Ocean. These relationships suggest that sites closer together have more similar fungal communities than sites that are more distant from each other. Previously, similar distance decay relationships were found present in other seagrass-associated fungal communities (40, 41). The observed distance decay relationship is likely driven by a combination of factors, including dispersal limitation, environmental filtering caused by local habitat differences, and priority effects. One limitation of this study is that local environmental conditions (e.g., temperature, salinity, dissolved oxygen) and biogeochemistry (e.g., sediment grain sizes, total organic carbon, carbon/nitrogen ratio) were not measured. Future research should incorporate environmental data to help identify the most important factors driving these patterns.

In addition to local environmental conditions, another factor that might lead to site-specific fungal community composition is host plant genetics. Host plant genotype has been found in other studies to strongly correlate with leaf fungal communities (63–65). The natural dispersal distance of *Z. marina* is thought to be less than 150 km, and there is some evidence of poor connectivity between locations and rampant inbreeding within locations (66–69). Given the strong population structure and weak dispersal of *Z. marina*, variation in *Z. marina* genotypes might be playing a role in structuring the fungal community differences observed here. However, it should be noted that Wainwright et al. failed to find a correlation between *S. isoetifolium* genetics and fungal community composition in their study (41). Regardless, there is growing evidence that seagrass-associated fungal communities are more similar at closer distances, and future work should look for correlations between environmental factors, *Z. marina* genetics, *Z. marina* dispersal, and the fungal community.

### Global mycobiome highlights importance of Chytridiomycota lineages.

The global mycobiome of *Z. marina* was generally composed of taxa previously observed to associate with *Z. marina* and other seagrass species by using culture-independent methods (see Fig. S14 in the supplemental material) (37–42). This diversity is also in line with cultivation efforts (37, 70, 71). All together, these results are consistent with the seagrass mycobiome being composed of many ASVs, including many Chytridiomycota lineages, for which a specific taxonomic assignment cannot be made based on current data sets that are biased toward terrestrial fungi (39, 40, 42). Likely contributing to this bias, only a few lineages of marine Chytridiomycota have been described using culture-based methods (10, 11), despite their dominance in DNA-based surveys of the marine environment (15, 42, 72). The inability to taxonomically classify fungal sequences is a persistent problem for studies of the marine environment generally and again serves to highlight the need for additional descriptive studies of marine fungi (15, 72–75).

### Small core mycobiomes are consistent with work in other seagrass species.

We were able to identify a small “common” core community associated with *Z. marina* tissues, with only a few ASVs unique to or shared between leaves and roots (Fig. 4). Previously, Trevathan-Tackett et al. (39) were able to identify a small core of eight fungal operational taxonomic units (OTUs) associated with the leaves of the close relative *Zostera muelleri*, in contrast to Hurtado-McCormick et al. (38), who were unable to identify a core fungal community on *Z. muelleri* leaves. The *Z. marina* core leaf and root mycobiomes were dominated by Sordariomycetes spp., Chytridiomycota lineages (including Lobulomycetaceae spp., which have previously been seen to dominate on this species [42]), and *Saccharomyces* spp. (Table 2; Tables S8 and S9). Sordariomycetes were also found to dominate the core leaf mycobiome in the study by Trevathan-Tackett et al. (39). Only four ASVs overlapped between the core communities for leaves, root, and sediment, and these ASVs were largely assigned to known ubiquitous marine generalists (e.g., *Cladosporium* [37] and *Malassezia* [51]).

### Considering model fit: are we underestimating the importance of deterministic processes?

The expected shape of a microbial abundance-occupancy distribution is an “S,” with abundant taxa having the highest occupancies and rare taxa having the lowest occupancies (50). However, the abundance-occupancy distributions for the data here do not have this shape. One possible reason for this deviation is an increased incidence of high-abundance but low-occupancy taxa (i.e., “clumping”) (76), which can be suggestive of niche selection (77). Clumping is thought to be affected by spatial variation in habitat quality, localized reproduction, and stochastic immigration-extinction processes (76). Clumping may also be the result of a competitive lottery-based assembly of the mycobiome (i.e., inhibitory priority effects), which means the first species to arrive will take over the entire niche, excluding other group members (78). In addition to not having the expected abundance-occupancy shape, the data had poor fit to the Sloan neutral model, although the fit was generally consistent with other studies of fungi (52) and also of bacteria (44). Thus, the poor fit of the neutral model may indicate that deterministic factors such as competition for niche space, extreme dispersal limitation, and variation in habitat quality may be playing a larger role than expected in assembly dynamics of seagrass-associated fungi.

### *Saccharomyces* sp. is an example of a globally distributed leaf epiphyte.

Both ITS_SV260 (Saccharomyces paradoxus) and 18S_SV928 (*Saccharomyces* sp.) were globally distributed and more abundant on leaves and roots (*P < *0.001) (Fig. S9 and S15). ITS_SV260 was predicted to be neutrally selected, while 18S_SV928 was predicted to be plant selected. S. paradoxus is a wild yeast, the sister species to Saccharomyces cerevisiae, and has been previously observed as a plant endophyte (79, 80). Given the relative abundance of *Saccharomyces* in both the ITS and 18S rRNA gene data sets on *Z. marina* leaves and roots, the global distribution of this taxon, and its deviation from the neutral model in the 18S rRNA gene data set (e.g., 18S_SV928), *Saccharomyces* sp. seems like a good candidate for future work in this system.

### *Lobulomyces* sp. is an example of a dispersal-limited leaf epiphyte.

In comparison, ITS_SV362 (*Lobulomyces* sp.) was found only at one site, dispersal limited, and is more abundant on leaves (*P < *0.001) (Fig. S10). Lobulomycetales have previously been observed in high abundance on and inside *Z. marina* leaves (42). Members of the Lobulomycetales and marine fungi more generally have been observed to have seasonal dynamics, which may relate to host dynamics and environmental conditions (81–84). Additionally, marine chytrids are known to parasitize seasonal blooms of diatoms (83, 85), and diatoms are the dominant eukaryotes observed on seagrass leaf tissues here (Fig. S11). Future studies should attempt to confirm whether these and other chytrids assigned to the core mycobiome of *Z. marina* are parasitizing closely associated diatoms or are associated with seagrass leaf tissues directly.

### *Colletotrichum* sp. appear to be endemic to only a few locations.

Previously, a *Colletotrichum* sp. ASV was found to be an abundant endophyte on and in leaves (42) and was later postulated to be a *Z. marina* specialist (37). In this study, no ASVs taxonomically assigned as *Colletotrichum* sp. were defined as part of the global core microbiome. However, one *Colletotrichum* sp. ASV, ITS_SV219, was found to deviate from the neutral model and was predicted to be dispersal limited. Its global distribution supports a pattern of endemism to only a few locations (Fig. S16). Local adaptation of marine fungi is consistent with patterns of endemism seen in terrestrial fungal studies (86, 87), and *Colletotrichum* sp. has been seen before as an endemic endophyte in Arabidopsis thaliana (88). One limitation of “core” community analyses generally is that it often underplays the importance of rare microbes which can also be essential for host function (89). Thus, future work should include studies of the functional importance of *Colletotrichum* sp. and other rare members of the *Z. marina* mycobiome.

### Fungi are not the dominant members of the *Z. marina*-associated eukaryotic community.

Fungi represented a mean relative abundance of less than 1% in the 18S rRNA amplicon data set. This is generally consistent with the proportion of fungi in other marine eukaryotic studies (e.g., 1.3% fungal sequences in a study by Hassett et al. [15]). Instead, the *Z. marina*-associated eukaryotic community was generally dominated by diatoms, oomycetes, and dinoflagellates. Diatom dominance was previously observed in a culture-independent study of *Z. marina* which found that the bacterial and eukaryotic epibiont communities are highly correlated (26). Additionally, oomycetes have been previously cultured in association with *Z*. *marina* and are thought to function as opportunistic pathogens or saprotrophs in this system (37, 90, 91).

### Functional predictions are consistent with plant-associated lifestyle.

Finally, we used FUNGuild to gain insight into possible functional roles of the mycobiome and found that the seagrass mycobiome is composed of a community of wood saprotrophs, ectomycorrhizal fungi, endophytic fungi, and plant pathogens. This functional distribution fits with what might be expected for a plant-associated community, as well as with what is known of the functional guilds of close relatives of fungal isolates previously isolated from *Z. marina* (37). However, many dominant members of the fungal community associated with *Z. marina* were not able to be assigned a functional guild, leaving much functional uncertainty still to be explored in this system. Additional studies characterizing seagrass-associated fungi are needed to understand the taxonomic diversity and functional roles of these fungi in the marine ecosystem generally and in particular when associated with seagrasses.

## MATERIALS AND METHODS

### Sample collection.

Samples were collected from 16 different globally distributed sites by researchers in the *Zostera* Experimental Network (ZEN) (Table 1) (92). Samples were collected subtidally at ∼1-m depth using a modified version of the collection protocol previously described by Fahimipour et al. (25). At each of the 16 sites, leaves and roots from individual *Z. marina* plants and adjacent sediment were collected for 12 individuals, resulting in a total of 576 samples (*n*_leaf_ = 192, *n*_root_ = 192, *n*_sediment_ = 192).

To obtain *Z. marina* leaf and root tissues for analysis here, researchers were instructed to (i) gently remove individual *Z. marina* plants from the sediment, (ii) briefly swish the individual in nearby seawater to remove loosely associated sediment from the roots, (iii) collect ∼5 roots and fully submerge them in a prelabeled 2-ml microcentrifuge tube filled with DNA/RNA Shield (Zymo Research, Inc., Irvine, CA, USA), and (iv) collect a 2-cm section of healthy green leaf tissue and fully submerge it in a prelabeled 2-ml microcentrifuge tube filled with DNA/RNA Shield. A sample of sediment was taken adjacent to each *Z. marina* individual from 1 cm under the sediment surface using a 6-ml (i.e., 6-cc) syringe. Briefly this was performed by (i) removing the plunger from the syringe, (ii) inserting the barrel of the syringe into the sediment, (iii) inserting the syringe plunger to form an airtight seal, (iv) removing the syringe from the sediment, (v) extruding the sediment until the base of the syringe plunger is at the 3-ml mark, and (vi) using an alcohol-sterilized plastic spatula to transfer ∼0.25 g of sediment into a prelabeled 2-ml microcentrifuge tube filled with DNA/RNA Shield. Samples were preserved in DNA/RNA Shield as it stabilizes DNA/RNA at room temperature. All samples were processed in the field immediately or within 5 h of collection. Samples subsequently were kept at room temperature and mailed to the University of California, Davis, within 2 weeks of sample collection.

### Molecular methods.

Samples were shipped from UC Davis to Zymo Research, Inc., for DNA extraction. Samples were transferred to 96-well plate format, with plates including both positive (ZymoBIOMICS microbial community standard) and negative (no-input) controls. DNA was extracted from samples using the ZymoBIOMICS DNA miniprep kit in accordance with the manufacturer’s protocol, with minor modifications as follows. Prior to DNA extraction, samples were heated at 65°C for 5 min to resuspend any white precipitate that had accumulated. Sediment samples were vortexed for 30 s to ensure homogenization and then, by use of a flame-sterilized spatula, transferred into ZR BashingBead lysis tubes until the tubes were two-thirds full. Leaf and root samples were vortexed for 30 s to dissociate any epiphytes, and then all the liquid was transferred into ZR BashingBead lysis tubes. For step 1, ZymoBIOMICS lysis solution was then added to samples such that the final volume was ∼1 ml. For step 2, samples were then subjected to a bead beater on the “homogenize” setting speed for 5 min. For step 4, 600 μl of supernatant was transferred to the filter tube. For step 11, only 50 μl of DNase/RNase free water was used for DNA elution. DNA concentrations for controls and a subset of samples per plate were first quantified with a Nanodrop (Thermo Fisher Scientific, Waltham, MA, USA), and subsequently all samples were quantified using Quant-iT PicoGreen (Thermo Fisher Scientific, Waltham, MA, USA). DNA was then shipped directly to the U.S. Department of Energy Joint Genome Institute (JGI) for amplicon sequencing.

### Sequence generation.

The ribosomal internal transcribed spacer 2 (ITS2) region was amplified via PCR using the fungus-specific ITS9F and ITS4R primer set (93, 94), and the 18S rRNA gene was amplified via PCR using the eukaryote-specific 565F and 948R primer set (95). The ITS region is the accepted universal fungal barcoding gene (96) and is made up of two subregions, ITS1 and ITS2. Historically, the ITS1 region has been used to survey fungal communities using short-read sequencing, but it has been shown to have greater length heterogeneity and result in lower phylogenetic richness than the ITS2 region (97–99). Additionally, it should be noted that ITS region primer choice can drastically affect the resulting community analyses (100, 101). The primer sets used here were selected and benchmarked by the JGI (102). Amplicon libraries were prepared according to the JGI’s iTag library construction standard operating protocol (SOP) v.1.0 (103). We briefly summarize the protocol here. Three replicate PCRs for each sample were performed in 96-well plate format with the following conditions: 94°C for 3 min, 35 cycles at 94°C for 25 s, 50°C for 60 s, 72°C for 90 s, and a final extension at 72°C for 10 min. After amplification, replicate PCR products were combined, and then samples were pooled based on DNA quantification of combined PCR replicates. Samples were then pooled at up to 184 samples per sequencing run and sequenced on an Illumina MiSeq (Illumina, Inc., San Diego, CA, USA) in 2 × 300 bp run mode. The resulting sequence data were demultiplexed by the JGI and processed through JGI’s quality-control system, which filters out known contaminant reads using the kmer filter in bbduk and also removes adaptor sequences (104). The quality-controlled sequence read files were downloaded and used for downstream analysis. The JGI iTag standard operating procedure does not include the sequencing of negative controls or blanks. For the ITS2 region, the raw data represented an average read depth of 236,610 with a range of 1 to 1,105,543 reads per sample and, for the 18S rRNA gene amplicon, an average read depth of 177,311 with a range of 3 to 1,095,911 reads per sample.

### Sequence processing.

Primers were removed using cutadapt (v. 2.1) (105). The resulting fastq files were analyzed in R (v. 4.0.2) using DADA2 (v. 1.12.1), phyloseq (v. 1.32.0), vegan (v. 2.5-6), microbiome (v. 1.10.0), ecodist (v. 2.0.5), EcoUtils (v. 0.1), DESeq2 (v. 1.28.1), ggplot2 (v. 3.3.2), tidyverse (v. 1.3.0), and many other R packages (106–144). For a detailed walkthrough of the following analysis using R, see the R-markdown summary file (145).

Prior to denoising in DADA2, reads were truncated at the first quality score of 2, and reads with an expected error greater than 2 were removed. Reads were then denoised and merged to generate tables of amplicon sequence variants (ASVs) using DADA2. Prior to downstream analyses, chimeric sequences were identified and removed from tables using removeBimeraDenovo (12.6% of sequences for ITS2 region, 4.5% of sequences for 18S rRNA gene). After chimera removal, samples had an average read depth of 120,270 with a range of 0 to 585,611 reads for the ITS2 region and an average read depth of 109,718 with a range of 0 to 616,884 reads for the 18S rRNA gene amplicon data. Taxonomy was inferred using the RDP Naive Bayesian Classifier algorithm with a modified UNITE (v. 8.2 “all eukaryotes”) database for ITS2 region sequences and the SILVA (v. 138) database for 18S rRNA gene sequences, resulting in 89,754 and 53,084 ASVs, respectively (146–149). The UNITE database was modified to include a representative ITS2 region amplicon sequence for the host plant, *Z. marina* (GenBank accession no. KM051458.1), as was done previously by Ettinger and Eisen (42). ASVs were then each assigned a unique name by giving each a number preceded by “ITS” or “18S” and then “SV,” which stands for sequence variant (e.g., ITS_SV1, ITS_SV2, etc., and 18S_SV1, 18S_SV2, etc.).

Based on the results of Pauvert et al. (150), ITS-x was not run on the ITS2 region ASVs. However, we removed all ASVs taxonomically assigned as nonfungal at the domain level (e.g., ASVs assigned to the host plant, *Z. marina*, or other eukaryotic groups or with no domain-level classification) from the ITS2 region ASV table prior to downstream analysis, resulting in a final table of 5,089 ASVs representing 488 samples (*n*_leaf_ = 179, *n*_root_ = 173, *n*_sediment_ = 136). A total of 88 samples were dropped from the analysis either because they had no sequences after being processed through DADA2 or no remaining sequences after removal of nonfungal ASVs. The remaining samples had an average read depth of 4,990 with a range of 2 to 82,870 reads.

For the 18S rRNA gene ASV table, we generated two different filtered data sets: (i) a fungus-only data set and (ii) a general eukaryotic data set. For data set i, we removed all nonfungal ASVs from the 18S rRNA gene ASV table prior to downstream analysis of the fungi in this data set, resulting in a table of 1,216 fungal ASVs representing 409 samples (*n*_leaf_ = 146, *n*_root_ = 144, *n*_sediment_ = 119). A total of 167 samples were dropped from the analysis either because they had no sequences after being processed through DADA2 or because they had no remaining sequences after removal of all ASVs classified as nonfungal. For data set ii, we removed ASVs taxonomically classified as noneukaryotic and also as being from embryophytes (e.g., *Z. marina*) from the 18S rRNA gene ASV table, resulting in a table of 36,582 eukaryotic ASVs representing 556 samples (*n*_leaf_ = 187, *n*_root_ = 187, *n*_sediment_ = 182). A total of 20 samples were dropped from the analysis either because they had no sequences after being processed through DADA2 or no remaining sequences after filtering ASVs. The remaining samples had an average read depth of 327 with a range of 2 to 8,470 reads.

### Sequence analysis and visualization.

To rarefy microbiome data is an ongoing scientific discussion (151–154), and here we use both alternative normalization techniques and rarefaction. For the majority of the analyses, after investigating library sizes and rarefaction curves and to maintain sufficient replication across all sites (Fig. S17), we utilized raw read counts, proportions, centered log-ratio, or Hellinger transformations on the data as appropriate when performing statistical analyses and generating visualizations. Centered log-ratio and Hellinger transformations were performed using the transform function in the microbiome R package. Centered log-ratio (clr) values are scale invariant such that the same ratio is obtained regardless of differences in read counts and thus were suggested as appropriate transformations for microbiome analysis by Gloor et al. (152). We used rarefaction approaches when calculating abundance-occupancy curves to define core taxa. We used rarefy_even_depth in the phyloseq R package to subsample to 1,000 and 100 reads without replacement, respectively, for the ITS2 region and 18S ASV tables (Fig. S17) by following the code described by Shade and Stopnisek (50). These depths were selected after examining rarefaction curves for both data sets to balance maximizing the number of sequences per sample and allowing for curve saturation where possible while also minimizing the number of samples removed for downstream analyses.

To assess alpha (i.e., within-sample) diversity between sample types (leaf, root, and sediment), the Shannon index of samples was calculated in ASV tables containing raw read counts using the estimate_richness function in the phyloseq R package. Raw read counts were used instead of normalizing the data by rarefying, as this kind of subsampling has been shown to be statistically inappropriate (151). To assess alpha diversity across each of the 16 collection sites (Table 1) and across oceans, we first split the data set into different sample types (leaf, root, and sediment), and then for each sample type, we calculated the Shannon index of samples. Kruskal-Wallis tests with 9,999 permutations were used to test for significant differences in alpha diversity across comparisons (sample type, site, or ocean). For comparisons in which the Kruskal-Wallis test resulted in a rejected null hypothesis (*P < *0.05), Bonferroni’s corrected *post hoc* Dunn tests were performed.

To assess beta (i.e., between-sample) diversity, we calculated several ecological metrics (Bray-Curtis dissimilarity, Aitchinson distance, Hellinger distance) by using the ordinate function in phyloseq and visualized them using principal-coordinate analysis. The Bray-Curtis dissimilarity is a widely used ecological metric in microbial analyses which calculates the compositional dissimilarity between samples (155). The Aitchison distance, which is the Euclidean distance of clr-transformed samples, is thought to be better than the Bray-Curtis dissimilarity because it is more stable to data subsampling and is also a true linear distance (152, 156). The Hellinger distance, which is the Euclidean distance of Hellinger distance-transformed data, is based on differences in the proportions of taxa and is thought to be a more ecologically relevant representation of the composition of taxa between samples than the Bray-Curtis dissimilarity, which is biased toward abundant taxa (157, 158).

To test for significant differences in mean centroids between categories of interest (i.e., sample type, site, ocean) for each ecological metric (Bray-Curtis dissimilarity, Aitchinson distance, Hellinger distance), we performed permutational multivariate analyses of variance (PERMANOVAs) with 9,999 permutations, and to account for multiple comparisons, we adjusted *P* values using the Bonferroni correction (159). We also tested for significant differences in mean dispersions between different categories of interest by using the betadisper and permutest functions from the vegan package in R with 9,999 permutations. The *post hoc* Tukey’s honestly significant difference (HSD) test was performed on betadisper results that resulted in a rejected null hypothesis (*P < *0.05) to identify which categories had mean dispersions that were significantly different.

To test for correlations between the community distances (Bray-Curtis dissimilarity, Hellinger distance) and geographic distances between samples, we first split the data by ocean and sample type and then calculated the geographical distances between samples using the Haversine formula, which accounts for the spherical nature of Earth using the distm function in the geosphere R package. We then performed Mantel tests using 9,999 permutations and generated Mantel correlograms using the mantel and mantel.correlog functions in the vegan R package. Mantel tests were repeated with exclusion of community distances when the geographic distance was zero to assess if patterns persisted in the absence of data from the same site. To further support Mantel test results, we performed multiple regression on distance matrices (MRM) between community distances and geographic distances using 9,999 permutations via the MRM function in the ecodist R package. The code to perform distance decay analyses was adapted from a report by Wainwright et al. (40).

To visualize global fungal community composition across sample types (leaf, root, and sediment), we transformed raw read counts to proportions and collapsed ASVs into taxonomic orders by using the tax_glom function in phyloseq and then removed orders with a mean proportion of less than 1%.

To examine the contribution of specific ASVs to fungal community composition, we used the DESeq2 R package on the raw read counts to examine the log_2_ fold change (differential abundance) of ASVs across sample types (leaf, root, sediment) in both data sets. We then visualized the global distribution of ASVs found to have significantly different differential abundances (Bonferroni corrected *P < *0.05). To do this, we transformed the raw read counts to proportions and then filtered each data set to include only the single ASV of interest by using prune_taxa in the phyloseq R package.

A core microbial community is usually defined as taxa that occur above an arbitrary detection threshold (e.g., greater than 1% relative abundance) and also above an arbitrary occupancy threshold (e.g., from 30% in a report by Ainsworth et al. [160] to 95% in a study by Huse et al. [161]). In an attempt to define “common” core leaf, root, and sediment mycobiomes (“common” as defined by Risely [162]), we used a more standardized approach by building abundance-occupancy curves and then calculating the rank contribution of specific ASVs to beta diversity (Bray-Curtis) to identify putative core ASVs by using code from Shade and Stopnisek (50). ASVs were predicted to be in the core by using the final percent increase in beta diversity described by Shade and Stopnisek (50) with a final percent increase equal to or greater than 10%. We then fit the Sloan neutral model (43) to the abundance-occupancy curves using the code provided by Burns et al. (44) to predict whether core taxa were selected for by the environment (e.g., by the host plant, *Z. marina*), dispersal limited, or neutrally selected.

To investigate the general composition of the eukaryotic community and assess what proportion of the eukaryotic community is taxonomically classified as fungal, we first transformed raw read counts from the 18S rRNA gene ASV table filtered to include all eukaryotes to proportions and collapsed ASVs into taxonomic phyla using the tax_glom function in phyloseq. For visualization purposes, we then removed phyla with a mean proportion of less than 0.1%. The average relative abundance of eukaryotic phyla was then calculated for each sample type (leaf, root, sediment).

To investigate possible functional roles of seagrass-associated fungi, FUNGuild (v. 1.1) was run on the taxonomic assignments of ASVs from both the ITS2 region and 18S rRNA gene data sets (163). FUNGuild searches the taxonomic assignments at the genus level against an online Guilds database containing taxonomic keywords and functional metadata (e.g., trophic level, guild, etc.) and FUNGuild assignments are given confidence rankings of “highly probable,” “probable,” or “possible.” To assess ecological guilds of high confidence, we first visualized all annotations that were ranked as “highly probable” in either data set. We then investigated functional guilds that were assigned to only highly abundant ASVs in the data. To assess this, we filtered both the ITS2 region and 18S rRNA gene ASV tables to include only ASVs with a mean abundance of greater than 0.1% and then visualized the data in R.

### Data availability.

The JGI quality-controlled sequence reads generated for the ITS2 region and the 18S rRNA gene were deposited in GenBank under BioProject ID PRJNA667465 (Sequence Read Archive [SRA] no. SRR12804623 to SRR12805321) and PRJNA667462 (SRA no. SRR12803303 to SRR12804019), respectively. Sequence reads are also available from the JGI Genome Portal (https://genome.jgi.doe.gov/portal/Popandseaspecies/Popandseaspecies.info.html).
