# Supplementary material for: Global Diversity and Biogeography of the Zostera marina Mycobiome
Source: Appl Environ Microbiol. 2021 May 26;87(12):e02795-20. doi: 10.1128/AEM.02795-20 (PMC8174750; doi:10.1128/AEM.02795-20)
Supplement: SUPPLEMENTAL FILE 1 — Supplemental material. Download aem.02795-20-s0001.pdf, PDF file, 1.1 MB [file aem.02795-20-s0001.pdf]

Supplementary Materials:

Title: Global diversity and biogeography of the *Zostera marina* mycobiome

Authors: Cassandra L. Ettinger[a,b#], Laura E. Vann[a,b,c*], Jonathan A. Eisen[a,b,d]

[a]Genome Center, University of California, Davis, CA, United States
[b]Department of Evolution and Ecology, University of California, Davis, CA, United States
[c]Department of Genomics and Bioinformatics, Novozymes, Davis, CA United States
[d]Department of Medical Microbiology and Immunology, University of California, Davis, Davis, CA, United States

#Address correspondence Cassandra L. Ettinger**,** clettinger@ucdavis.edu

***Present address:** Department of Genomics and Bioinformatics, Novozymes, Davis, CA United States

Running title: Global seagrass mycobiome

Keywords: seagrasses, *Zostera marina*, marine fungi, microbial eukaryotes, 18S rRNA, ITS2, eelgrass, mycobiome, core, abundance-occupancy, dispersal-limited, plant-selected, global distribution

Figure S1. Community structure varies between sites and oceans. Principal coordinates analysis (PCoA) visualization of Hellinger distances of fungal communities associated with leaves, roots and sediment. For (A) ITS2 region amplicon data and (B) 18S rRNA amplicon data, points in the ordinations are colored by site collected (Table 2) and represented by shapes based on sample type (circles), root (triangles) or sediment (squares).

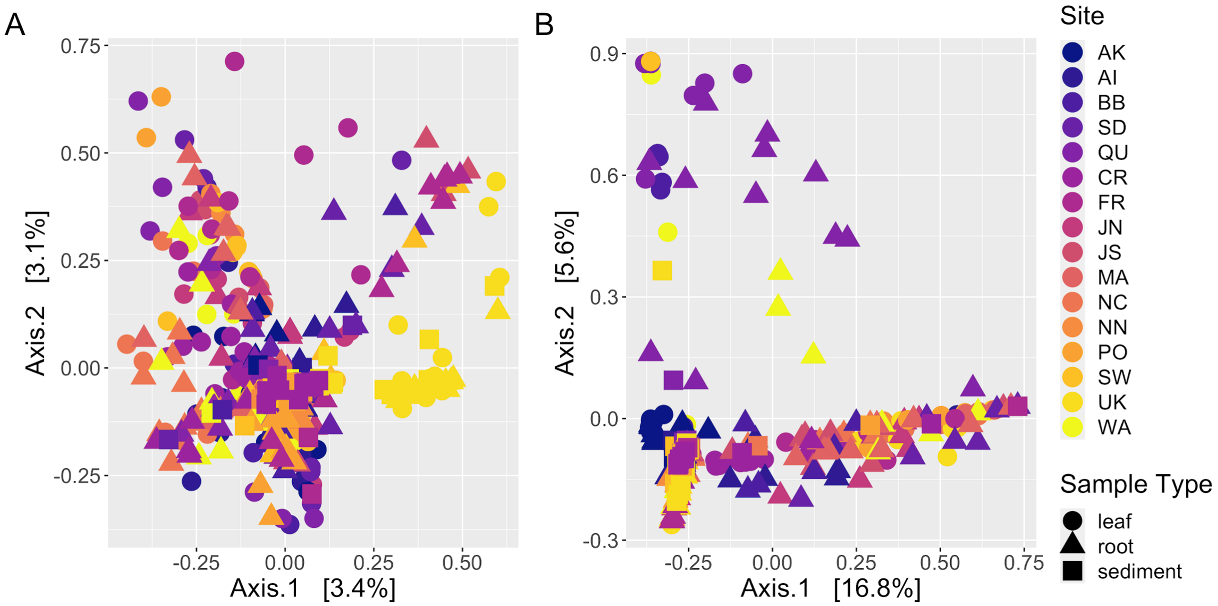

Figure S2. Mantel tests suggest a distance-decay relationship. Scatterplots depicting the weak, but significant positive distance–decay relationship between fungal community beta diversity (Hellinger distance) using the 18S rRNA gene amplicon data and geographical distance (km) between sites for leaves from the (A) Pacific Ocean, and (B) Atlantic Ocean, roots from the (C) Pacific Ocean, and (D) Atlantic Ocean, and sediment from the (E) Pacific Ocean and (F) Atlantic Ocean.

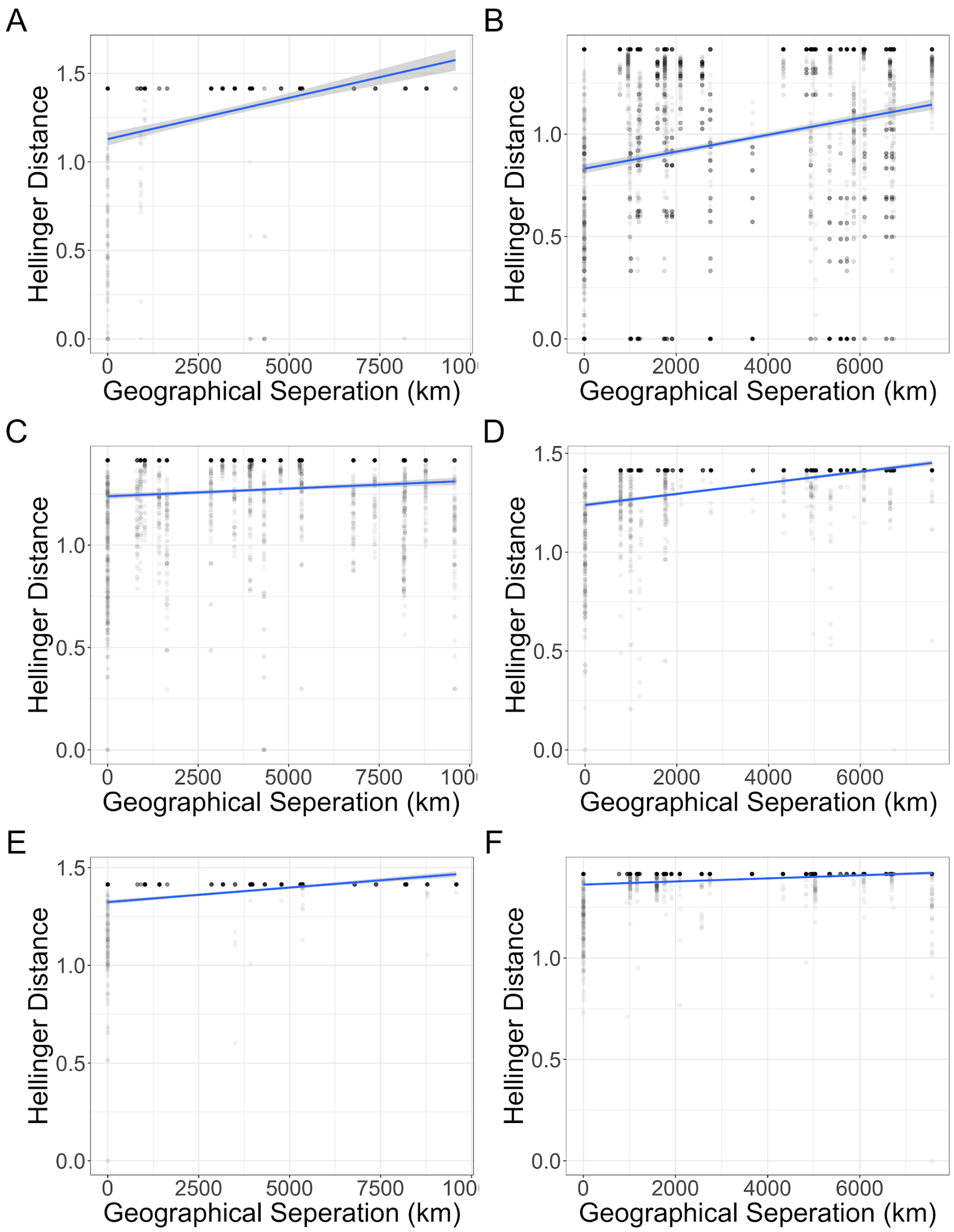

A
B
C
D
E
F
Hellinger Distance
Geographical Seperation (km)
0.0
0.5
1.0
1.5
0
2500
5000
7500
2000
4000
6000

Figure S3. Mantel correlograms suggest distance-decay relationships are driven by samples from closest geographic sites. Mantel correlograms for the ITS2 region amplicon data for the (A) Pacific Ocean, and (B) Atlantic Ocean. The x-axis is binned geographic distance class indices and the y-axis is the Mantel correlation statistic. Black points are statistically significant ($p < 0.05$) and white points are not statistically significant.

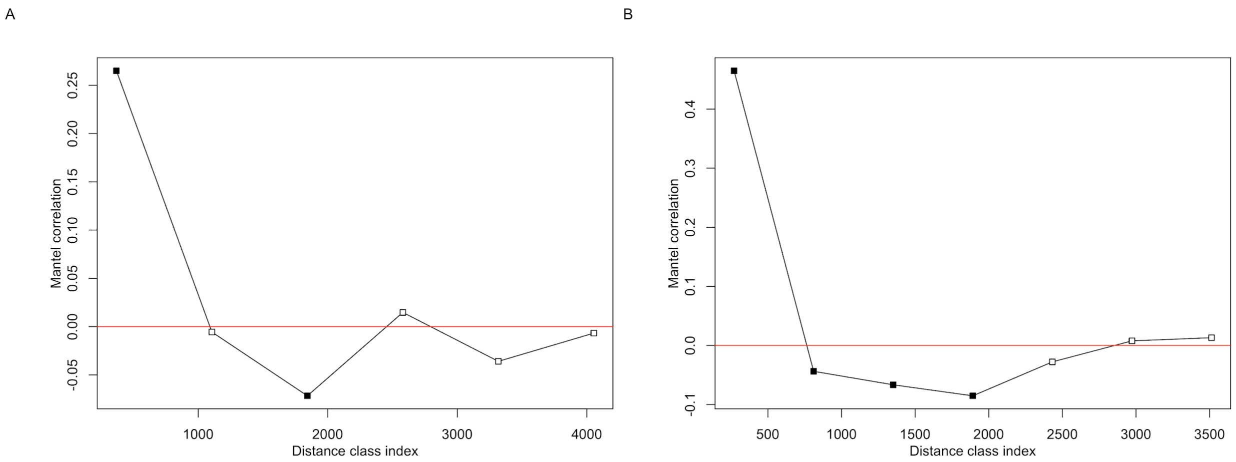

Figure S4. Mantel tests suggest a distance-decay relationship. Scatterplots depicting the weak, but significant positive distance–decay relationship between fungal community beta diversity (Hellinger distance) using the ITS2 region amplicon data and geographical distance (km) between sites for roots from the (A) Pacific Ocean, and (B) Atlantic Ocean, and sediment from the (C) Pacific Ocean, and (D) Atlantic Ocean.

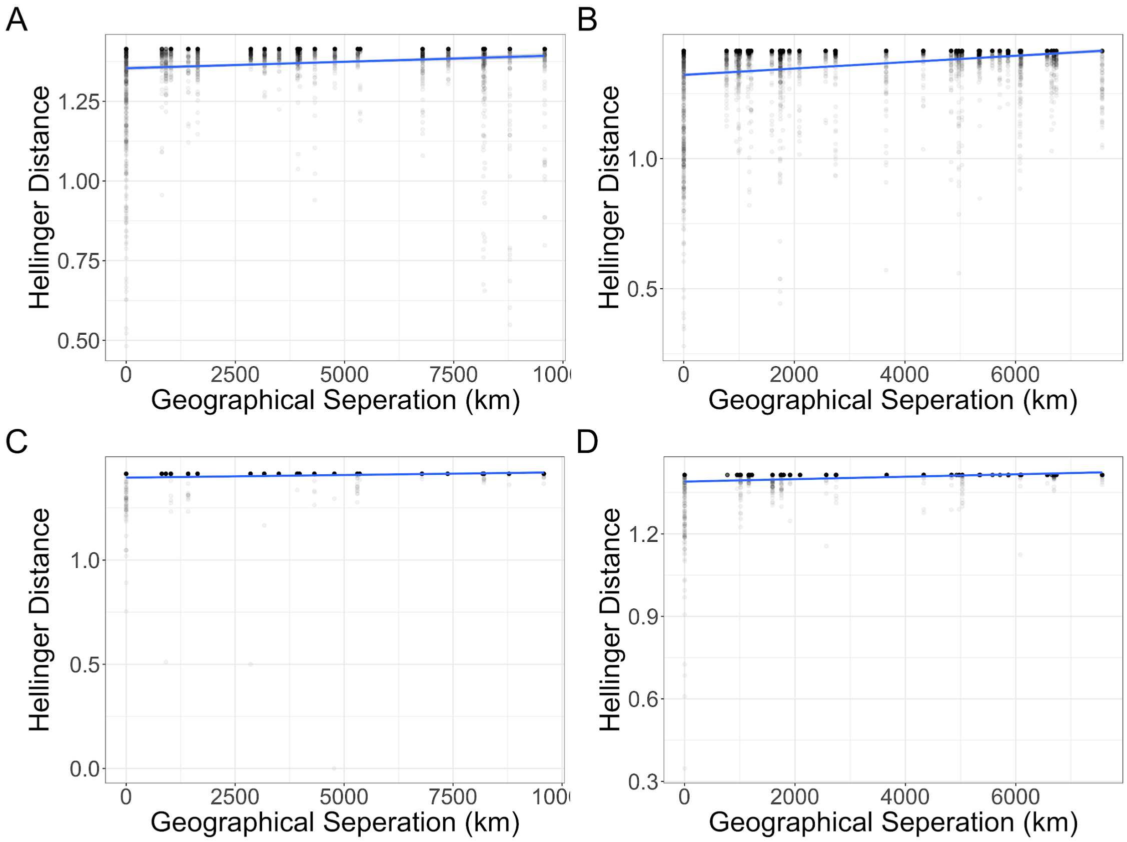

Figure S5. Overlap between mycobiomes of individual *Z. marina* tissues. Venn diagrams representing shared ASVs for each sample type (leaf, root, sediment) for (A) ITS2 region amplicon data, and (B) 18S rRNA gene amplicon data.

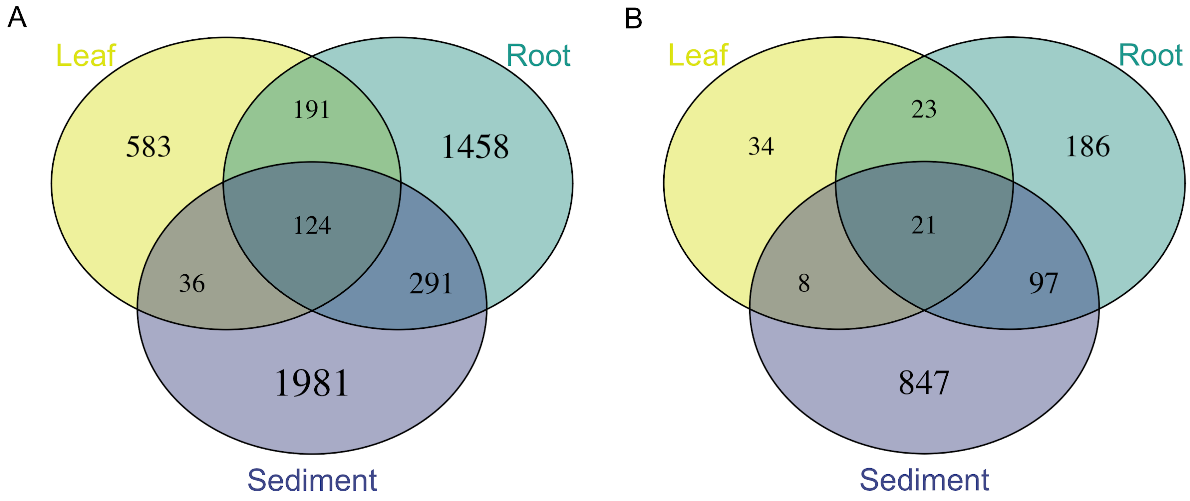

Figure S6. Abundance-occupancy distributions reveal core mycobiomes. Abundance-occupancy distributions were used to define core members of the (A) leaf, (B) root and (C) sediment mycobiomes for the 18S rRNA gene amplicon data. Each point represents an ASV with core members indicated by a color (leaf = yellow, root = green, sediment = blue) and non-core ASVs in white. Ranked ASVs were predicted to be in the core based on a final percent increase of equal or greater than 10%. The solid line represents the fit of the neutral model, and the dashed line is 95% confidence around the model prediction. ASVs above the neutral model are predicted to be selected for by the environment (e.g. by the host plant, *Z. marina*), and those below the model are predicted to be selected-against or dispersal-limited.

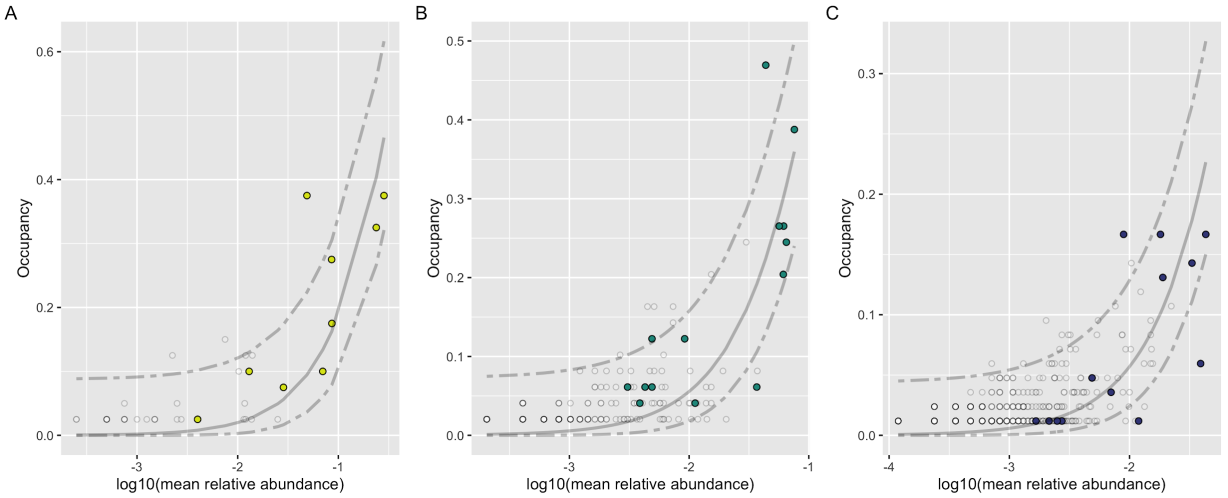

Figure S7. Differentially abundant ASVs across tissues. Fungal ITS2 region ASVs were identified using DESeq2 whose abundance differed significantly between pair-wise sample types (leaf, root, sediment). Each plot shows the log_2 fold change of ASVs which were differentially abundant between (A) leaves and rhizosphere sediment, (B) leaves and roots, and (C) roots and rhizosphere sediment. A positive log_2 fold change means the ASV was more abundant in the first tissue and a negative log_2 fold change means the ASV was more abundant in the second tissue in the comparison.

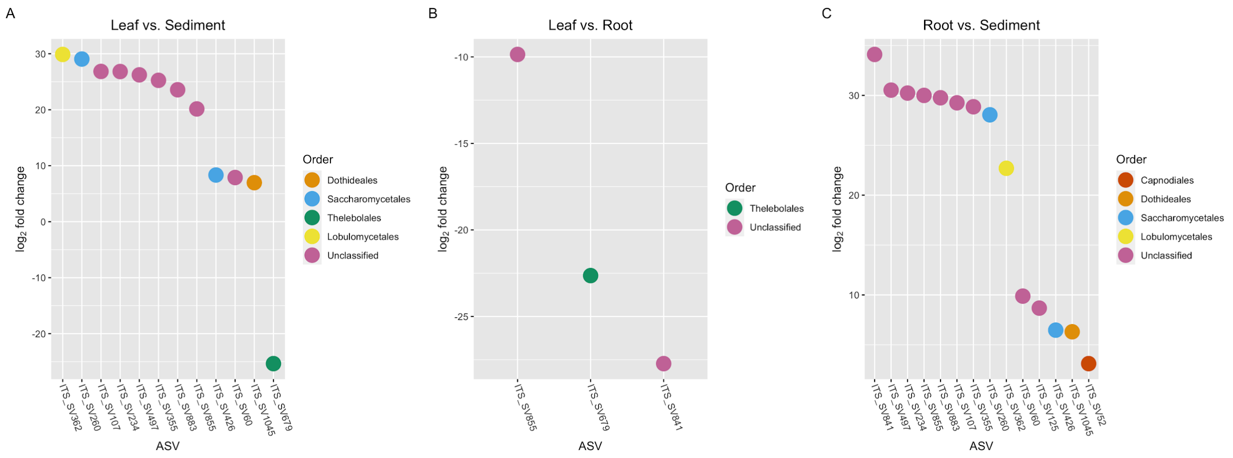

Figure S8. Differentially abundant ASVs across tissues. Fungal 18S rRNA gene ASVs were identified using DESeq2 whose abundance differed significantly between pair-wise sample types (leaf, root, sediment). Each plot shows the $\log_2$ fold change of ASVs which were differentially abundant between (A) leaves and rhizosphere sediment, and (B) roots and rhizosphere sediment. A positive $\log_2$ fold change means the ASV was more abundant in the first tissue and a negative $\log_2$ fold change means the ASV was more abundant in the second tissue in the comparison. No ASVs were differentially abundant between leaves and roots.

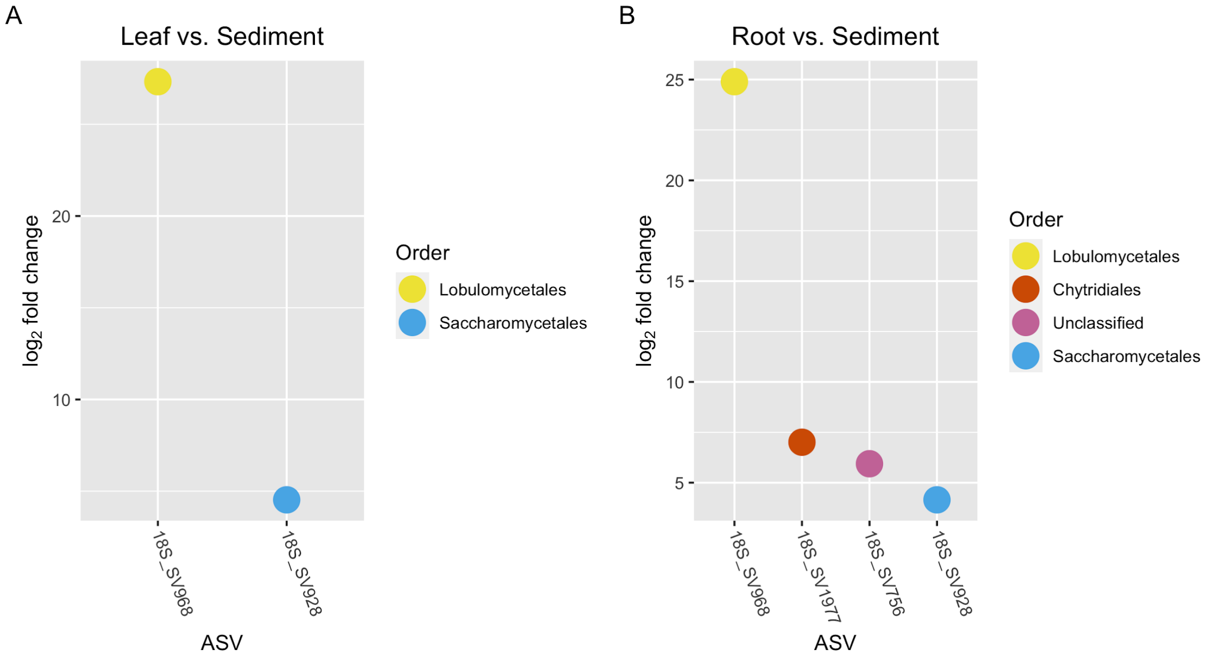

Figure S9. Example of differentially abundant neutrally selected core ASV. Here we show the global distribution of ITS_SV260, an ASV predicted to be a neutrally selected member of the core mycobiomes of both leaves and roots and also differentially abundant between leaves and sediment ($p < 0.001$**)**, and roots and sediment ($p < 0.001$) using DESeq2. In (A) we plot the mean relative abundance of ITS_SV260 at each site on leaves on a global map, and in (B) we plot the mean relative abundance of ITS_SV260 on leaves, roots and sediment across sites, with the standard error of the mean represented by error bars and bars colored by sample type.

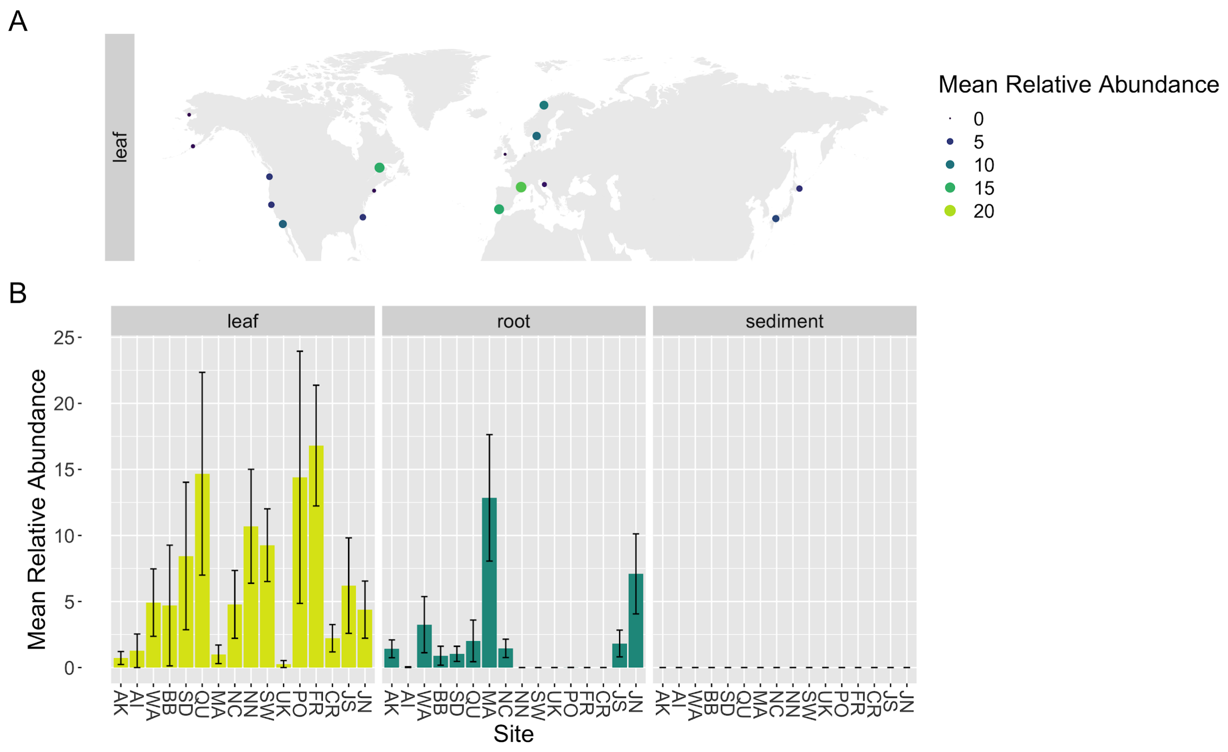

Figure S10. Example of differentially abundant dispersal-limited core ASV. Here we show the global distribution of ITS_SV362, an ASV predicted to be dispersal-limited and a member of the core mycobiomes of leaves and also differentially abundant between leaves and sediment ($p < 0.001$), and roots and sediment ($p < 0.001$) using DESeq2. In (A) we plot the mean relative abundance of ITS_SV362 at each site on leaves on a global map, and in (B) we plot the mean relative abundance of ITS_SV362 on leaves, roots and sediment across sites, with the standard error of the mean represented by error bars and bars colored by sample type.

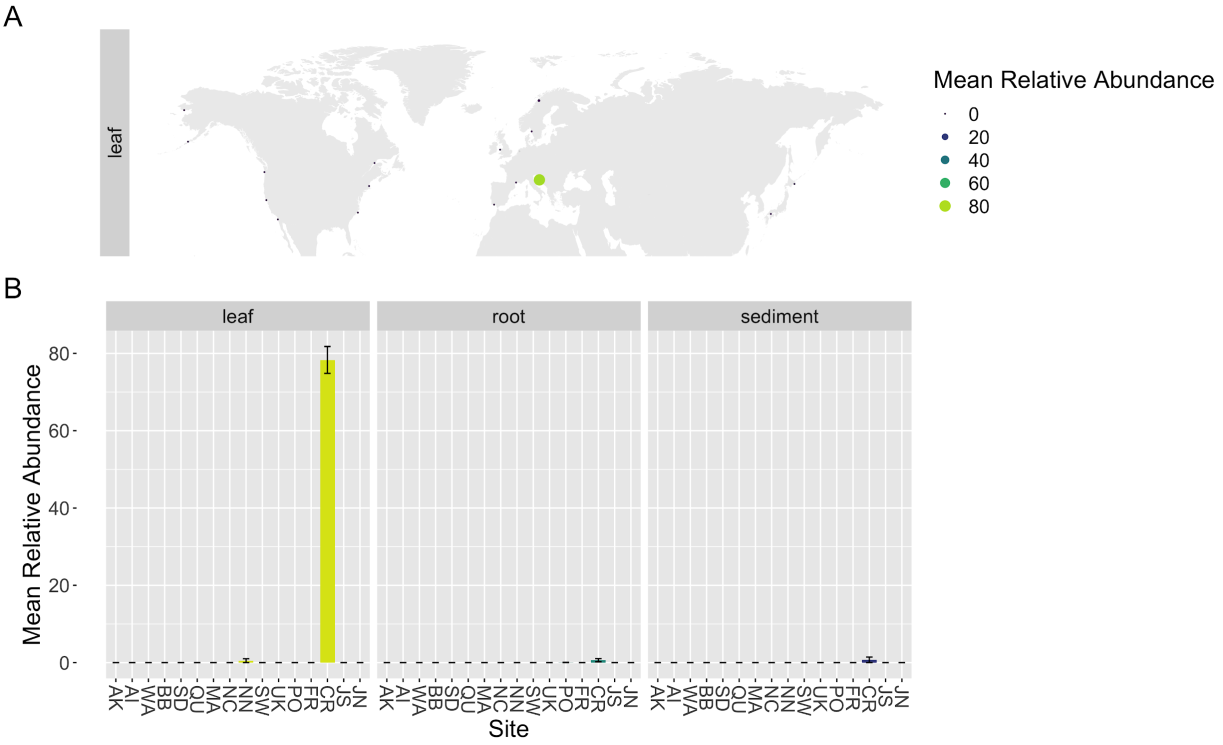

Figure S11. Eukaryotic community composition differs between tissues. The mean relative abundance of broad taxonomic groups are shown for the 18S rRNA gene amplicon data. Only groups with a mean relative abundance of greater than 0.1 percent are shown across sample types (leaf, root and sediment), with the standard error of the mean represented by error bars and bars colored by taxonomic groups.

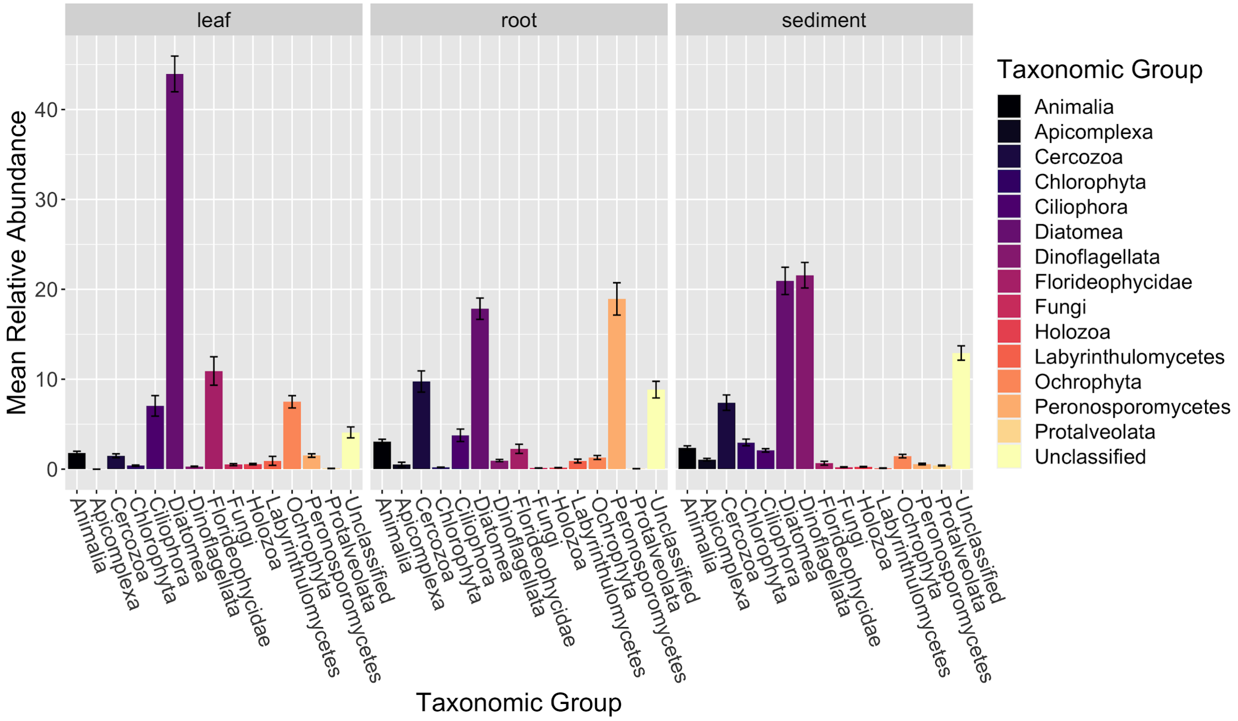

Figure S12. Counts of fungal trophic modes based on the ITS2 region amplicon data. The count of fungal trophic modes based on FUNGuild, (A) across fungal guilds assigned with high probability, and (B) across all probability levels for ASVs with a mean relative abundance of 0.1 percent or greater.

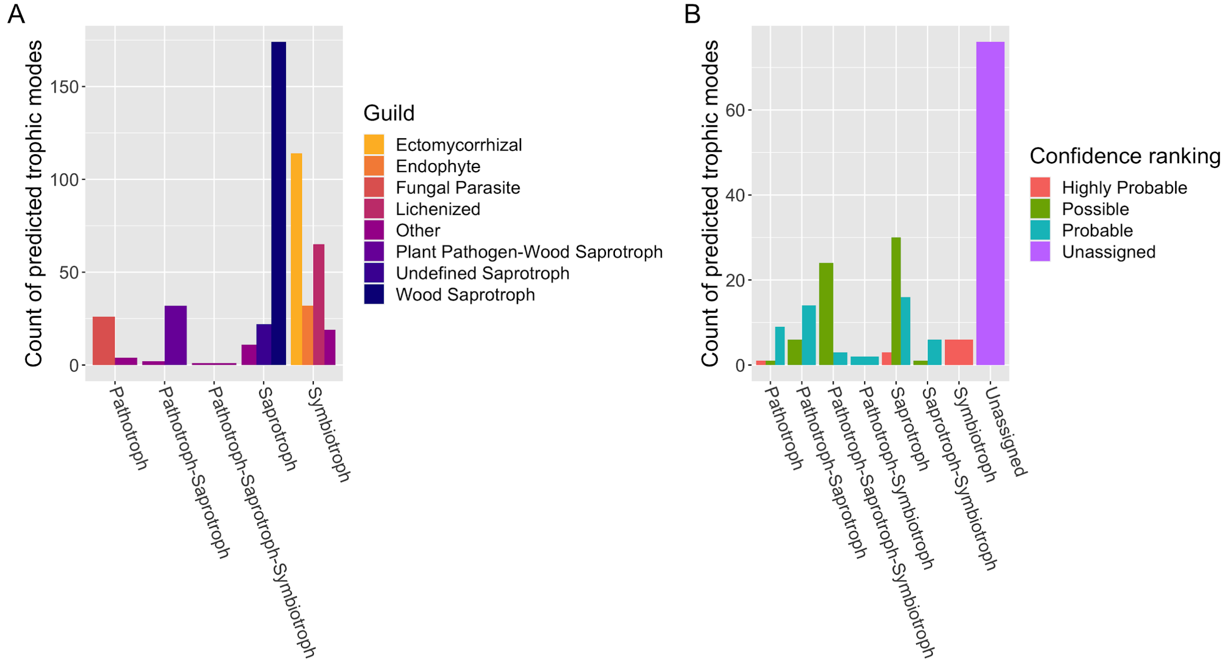

Figure S13. Counts of fungal trophic modes based on the 18S rRNA gene amplicon data. The count of fungal trophic modes based on FUNGuild, (A) across fungal guilds assigned with high probability, and (B) across all probability levels for ASVs with a mean relative abundance of 0.1 percent or greater.

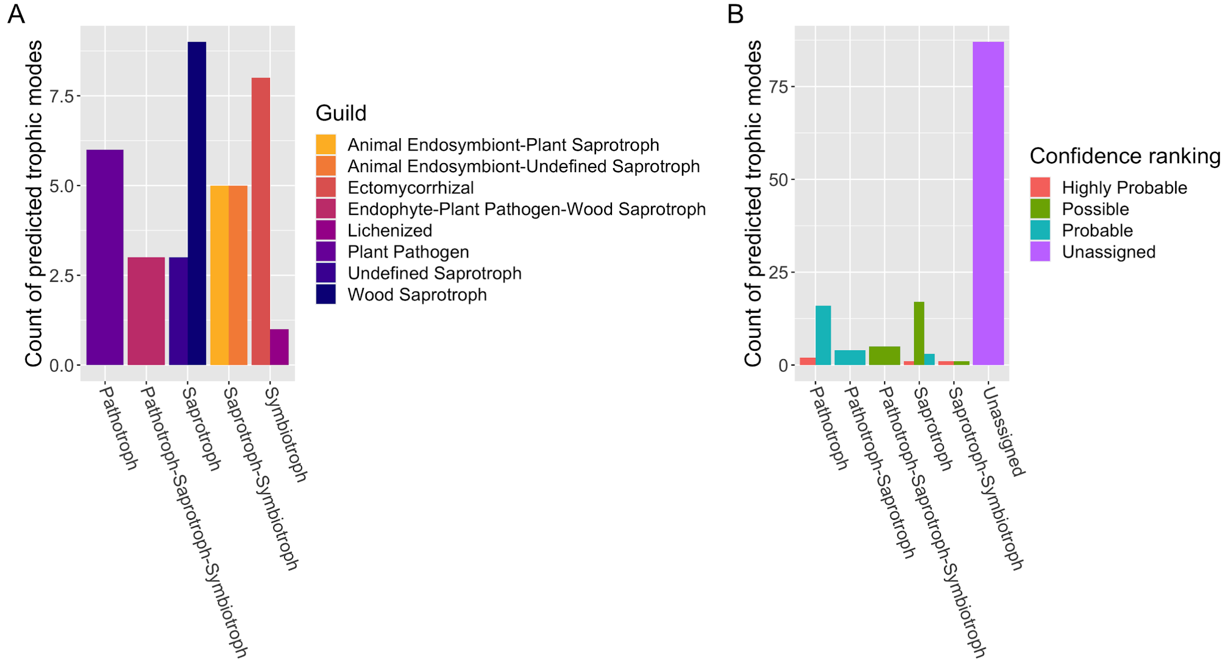

Figure S14. Fungal community composition differs between tissue types. The mean relative abundance of taxonomic orders are shown for the (A) ITS2 region amplicon data and (B) the 18S rRNA amplicon data. Only orders with a mean relative abundance of greater than one percent are shown across bulk sample types (leaf, root, and sediment), with the standard error of the mean represented by error bars and bars colored by taxonomic phylum.

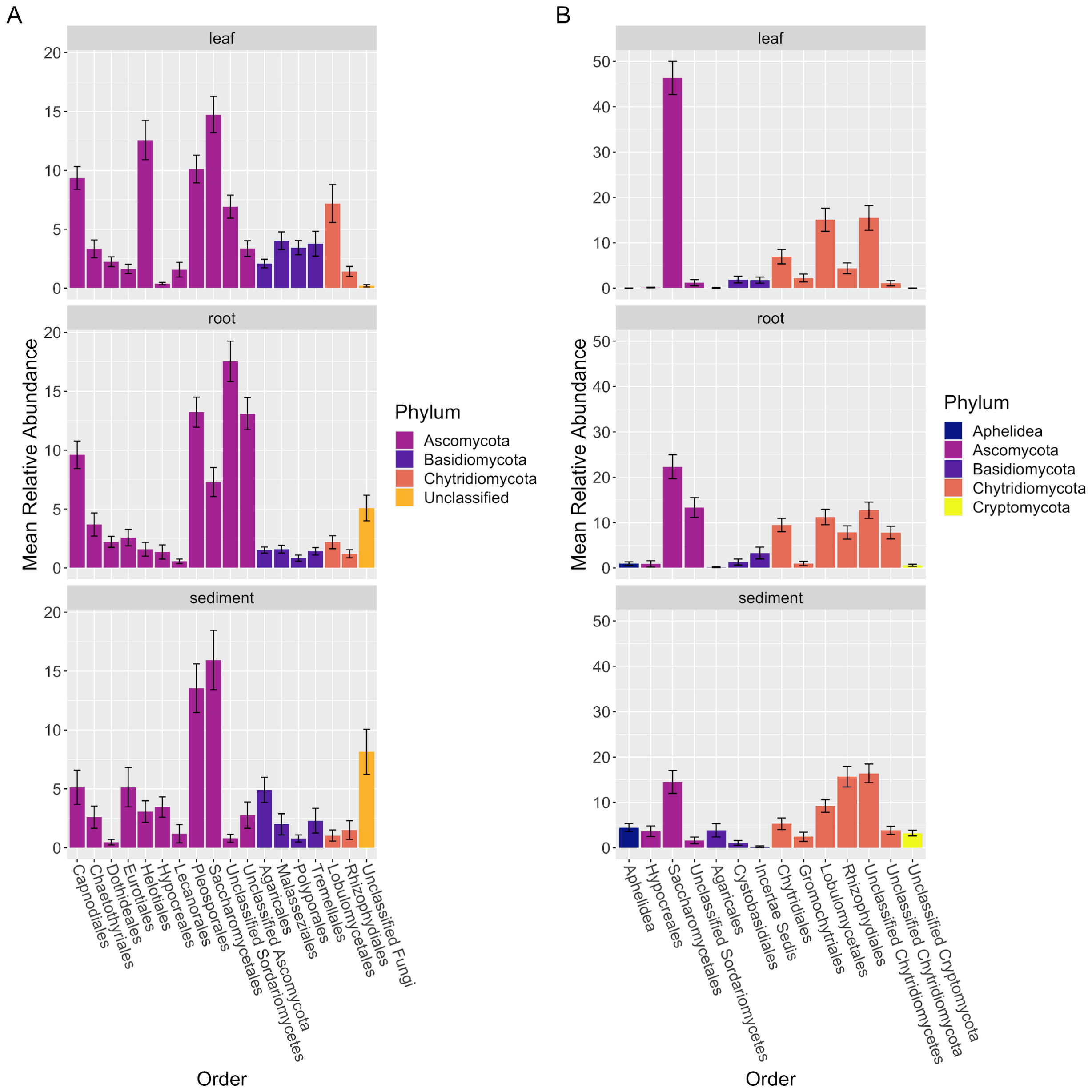

Figure S15. Example of differentially abundant plant-selected core ASV. Here we show the global distribution of 18S_SV928, an ASV predicted to be plant-selected and a member of the core mycobiomes of both leaves and roots and also differentially abundant between leaves and sediment ($p < 0.001$**)**, and roots and sediment ($p < 0.001$) using DESeq2. In (A) we plot the mean relative abundance of 18S_SV928 at each site on leaves on a global map, and in (B) we plot the mean relative abundance of 18S_SV928 on leaves, roots and sediment across sites, with the standard error of the mean represented by error bars and bars colored by sample type.

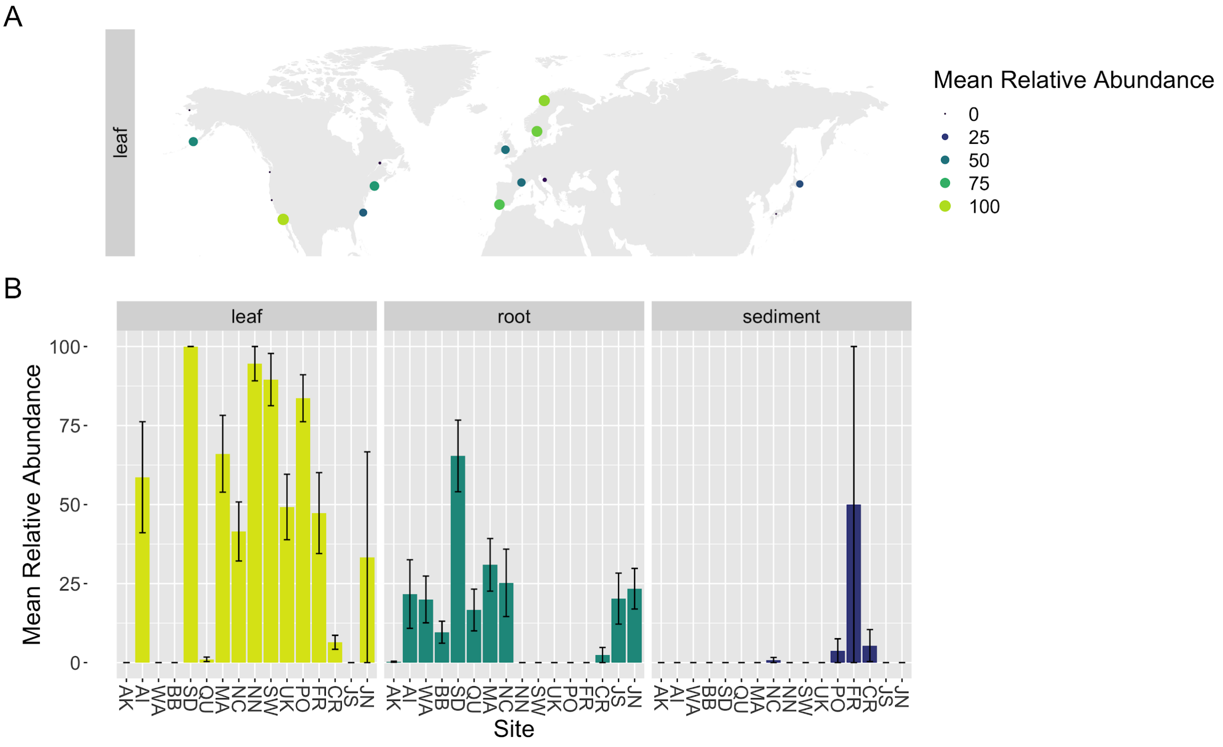

Figure S16. Example of dispersal-limited ASV. Here we show the global distribution of ITS_SV219, taxonomically identified as a *Colletotrichum sp*. and predicted to be dispersal-limited. In (A) we plot the mean relative abundance of ITS_SV219 at each site on leaves on a global map, and in (B) we plot the mean relative abundance of ITS_SV219 on leaves, roots and sediment across sites, with the standard error of the mean represented by error bars and bars colored by sample type.

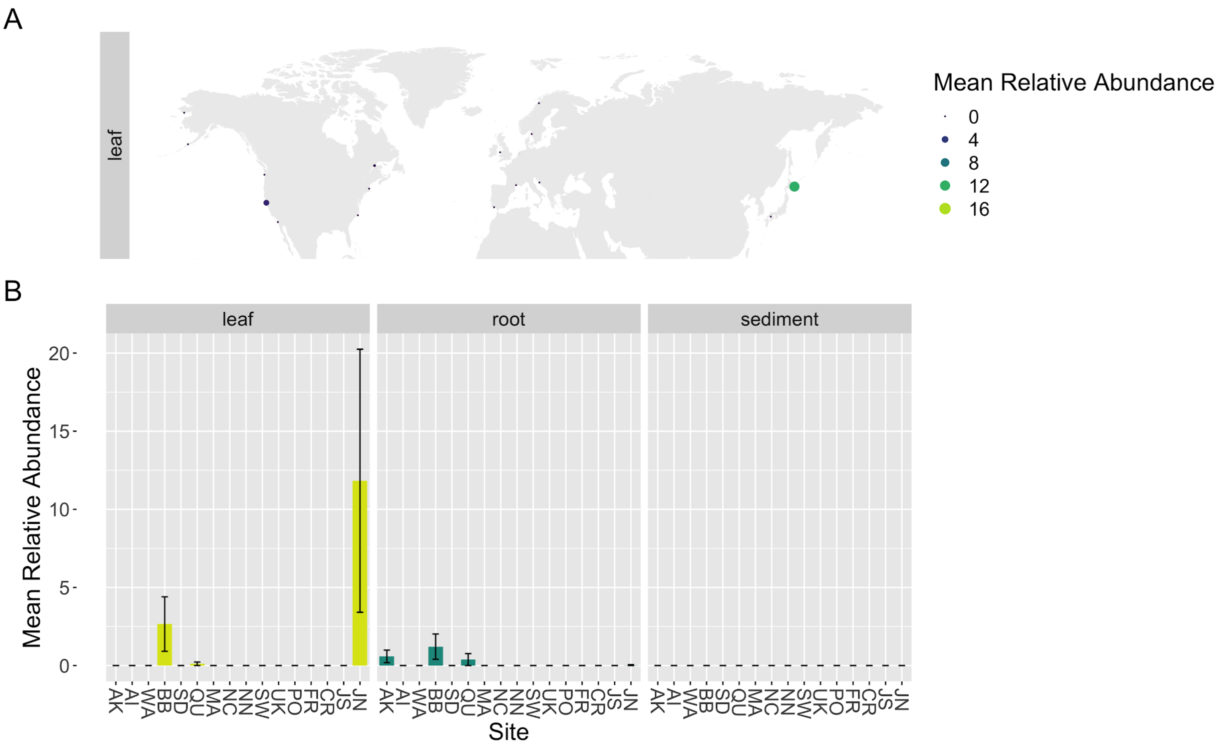

Figure S17. Rarefaction curves and library sizes for ITS2 region and 18S rRNA gene region amplicon data. Shown here are rarefaction curves portraying the relationship between the number of observed ASVs (species richness) and the number of reads (sample size) for the (A) ITS2 region, and (B) 18S rRNA gene amplicon data. Lines are colored by sample type (leaf = yellow, root = green, sediment = blue). Additionally, a zoomed in version of the rarefaction curves depicted in (A) and (B) are shown for the (C) ITS2 region (x-axis cut-off after 2000 reads) and (D) 18S rRNA gene amplicon data (x-axis cut-off after 200 reads). Finally, also shown are histograms of the frequency of samples that have $\log_{10}$ transformed read counts using a bin width of one for the (E) ITS2 region, and (F) 18S rRNA gene amplicon data. Bars are colored by sample type (leaf = yellow, root = green, sediment = blue). Orange vertical lines represent the sample depth at which subsampling was performed for core analyses for each dataset. For (E) this is at a $\log_{10}$ transformed read count of three (representing 1000 reads) and for (F) this is at a $\log_{10}$ transformed read count of two (representing 100 reads).

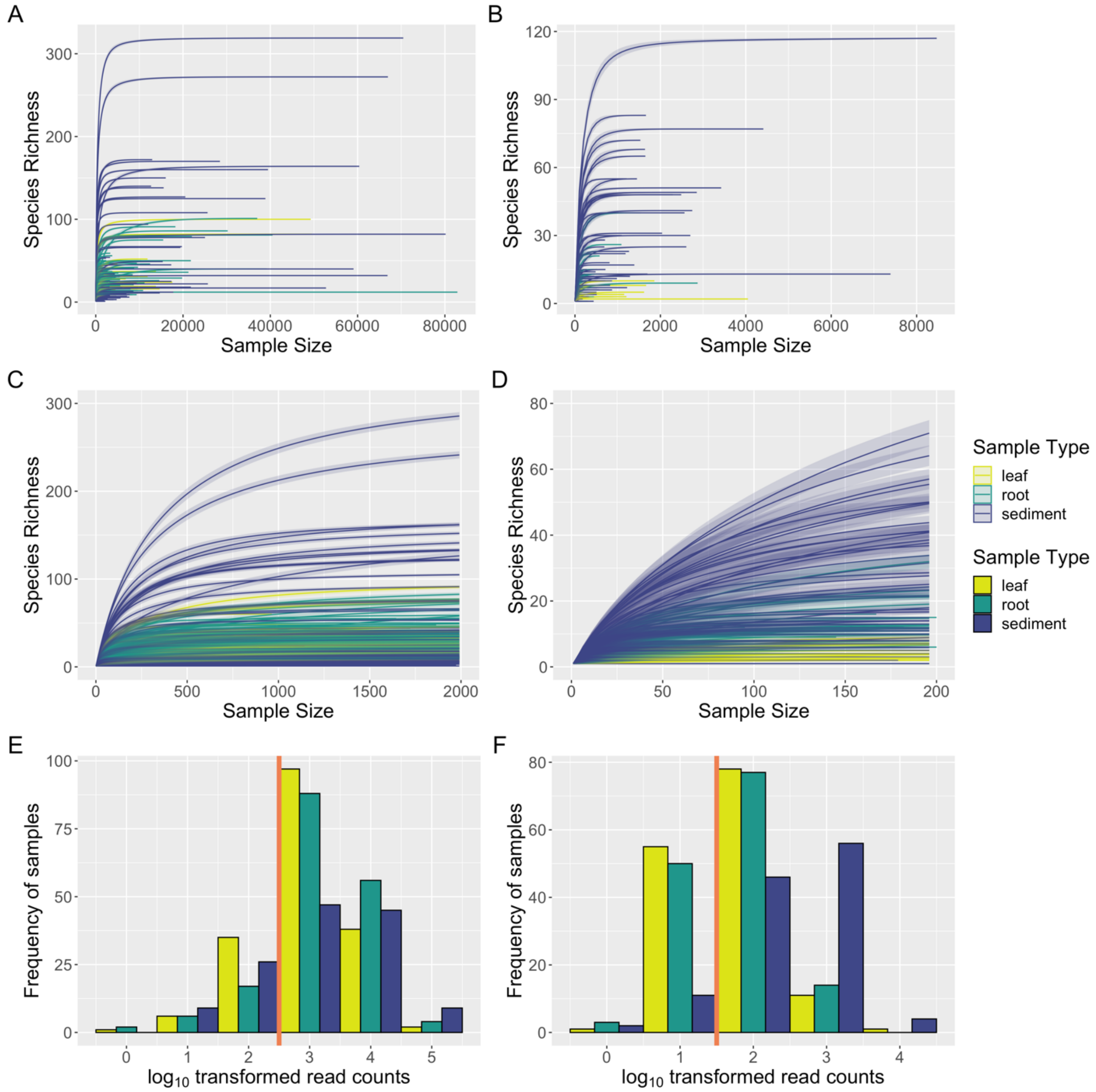
A
B
C
D
E
F
Species Richness
Sample Size
Frequency of samples
log10 transformed read counts
Sample Type
leaf
root
sediment
Sample Type
leaf
root
sediment

Table S1. Pair-wise *post hoc* Dunn tests of leaf Shannon diversity in the ITS2 region dataset. Kruskal–Wallis tests identified significant differences in alpha diversity in leaves between sites ($p < 0.001$, Figure 1B). Here we report the results of Bonferroni corrected *post hoc* Dunn tests which were used to assess which pair-wise site comparisons were driving observed differences. Pair-wise comparisons that are significantly different are bolded ($p < 0.05$) and shaded gray. Full site names can be found in Table 2.

	AK	WA	BB	SD	QU	MA	NC	NN	SW	UK	PO	FR	CR	JS	JN
AI	0.5886	0.0880	0.9193	**0.0296**	0.0625	0.4334	0.1403	0.6312	0.3771	0.3947	0.2788	0.3457	**0.0045**	0.3296	0.9086
AK		0.2755	0.7138	**0.0048**	**0.0125**	0.1886	0.3778	0.3396	0.7200	0.7356	0.0890	0.1182	**0.0005**	0.6463	0.6552
WA			0.1663	**0.0001**	**0.0003**	**0.0122**	0.8277	**0.0392**	0.4434	0.4405	**0.0044**	**0.0053**	**0.0001**	0.5058	0.1126
BB				**0.0385**	0.0758	0.4328	0.2402	0.5919	0.4932	0.5047	0.2811	0.3400	**0.0078**	0.4368	0.9843
SD					0.7630	0.2035	**0.0001**	0.1417	**0.0012**	**0.0017**	0.3633	0.2797	0.5827	**0.0007**	**0.0218**
QU						0.3422	**0.0005**	0.2522	**0.0040**	**0.0045**	0.5242	0.4366	0.4077	**0.0028**	**0.0494**
MA							**0.0220**	0.8207	0.0872	0.0933	0.7494	0.8658	0.0599	0.0653	0.3924
NC								0.0567	0.5877	0.5924	**0.0073**	**0.0100**	**0.0001**	0.6459	0.1740
NN									0.1808	0.1936	0.5830	0.6783	**0.0418**	0.1457	0.5785
SW										0.9860	**0.0385**	**0.0486**	**0.0001**	0.9164	0.4288
UK											**0.0432**	0.0575	**0.0002**	0.9124	0.4355
PO												0.8967	0.1332	**0.0298**	0.2378
FR													0.0863	**0.0396**	0.2879
CR														**0.0001**	**0.0035**
JS															0.3723

Table S2. Pair-wise *post hoc* Dunn tests of leaf Shannon diversity in the 18S rRNA gene dataset. Kruskal–Wallis tests identified significant differences in alpha diversity in leaves between sites ($p < 0.001$, Figure 1D). Here we report the results of Bonferroni corrected *post hoc* Dunn tests which were used to assess which pair-wise site comparisons were driving observed differences. Pair-wise comparisons that are significantly different are bolded ($p < 0.05$) and shaded gray. Full site names can be found in Table 2.

	AK	WA	BB	SD	QU	MA	NC	NN	SW	UK	PO	FR	CR	JS	JN
AI	**0.0024**	0.4100	0.0667	0.7785	0.5666	0.5624	**0.0447**	0.7823	0.7608	0.0610	0.7628	0.2240	**0.0481**	0.9934	0.7096
AK		0.0691	0.3806	**0.0216**	**0.0069**	**0.0062**	0.2785	**0.0001**	**0.0001**	0.2561	**0.0029**	0.0687	0.2632	**0.0092**	**0.0057**
WA			0.4261	0.3786	0.7709	0.7656	0.3974	0.2172	0.2004	0.4334	0.5802	0.8707	0.4123	0.4615	0.2860
BB				0.1066	0.1553	0.1566	0.9806	**0.0155**	**0.0140**	0.9661	0.1054	0.4886	0.9892	0.1054	0.0666
SD					0.4642	0.4643	0.1060	0.8681	0.8863	0.1077	0.5533	0.2644	0.1069	0.7975	1.0000
QU						0.9962	0.1071	0.3041	0.2816	0.1273	0.8634	0.4874	0.1041	0.6229	0.3921
MA							0.1057	0.3005	0.2830	0.1289	0.8585	0.4920	0.1056	0.6211	0.3973
NC								**0.0054**	**0.0078**	0.9828	0.0683	0.4549	0.9864	0.0965	0.0609
NN									0.9952	**0.0073**	0.4451	0.0811	**0.0053**	0.8727	0.8582
SW										**0.0065**	0.4221	0.0723	**0.0059**	0.8618	0.8655
UK											0.0818	0.4889	0.9875	0.1048	0.0687
PO												0.3736	0.0672	0.7685	0.4678
FR													0.4640	0.3099	0.1676
CR														0.1021	0.0634
JS															0.7641

Table S3. Pair-wise *post hoc* Dunn tests of root Shannon diversity in the ITS2 region dataset. Kruskal–Wallis tests identified significant differences in alpha diversity in leaves between sites ($p < 0.001$, Figure 1B). Here we report the results of Bonferroni corrected *post hoc* Dunn tests which were used to assess which pair-wise site comparisons were driving observed differences. Pair-wise comparisons that are significantly different are bolded ($p < 0.05$) and shaded gray. Full site names can be found in Table 2.

	AK	WA	BB	SD	QU	MA	NC	NN	SW	UK	PO	FR	CR	JS	JN
AI	0.0898	0.0646	**0.0083**	0.4736	0.6015	0.2357	**0.0017**	0.0755	0.3773	**0.0179**	0.2091	0.7918	0.8138	0.2962	0.7836
AK		0.9659	0.3766	0.3656	0.2911	0.6487	0.2091	0.9527	0.4687	0.6446	0.6675	0.1843	0.0670	0.5633	0.1690
WA			0.3705	0.3129	0.2395	0.5933	0.2117	0.9758	0.4303	0.6488	0.6472	0.1550	0.0530	0.5115	0.1373
BB				0.0609	0.0506	0.1546	0.7887	0.4095	0.0944	0.6396	0.1852	**0.0212**	**0.0073**	0.1268	**0.0189**
SD					0.8651	0.6528	**0.0196**	0.3301	0.8470	0.1367	0.6320	0.6554	0.3813	0.7452	0.6461
QU						0.5702	**0.0143**	0.2521	0.7419	0.0912	0.5153	0.7807	0.4776	0.6445	0.7874
MA							0.0579	0.5927	0.7985	0.3014	0.9319	0.3807	0.1801	0.9083	0.3762
NC								0.2368	**0.0387**	0.4567	0.0786	**0.0075**	**0.0030**	**0.0480**	**0.0060**
NN									0.4400	0.6435	0.6408	0.1626	0.0601	0.5169	0.1520
SW										0.2042	0.7398	0.5635	0.2902	0.8851	0.5661
UK											0.3674	0.0559	**0.0178**	0.2414	**0.0486**
PO												0.3668	0.1621	0.8393	0.3552
FR													0.6618	0.4588	0.9803
CR														0.2179	0.6467
JS															0.4527

Table S4. Pair-wise *post hoc* Dunn tests of root Shannon diversity in the 18S rRNA gene dataset. Kruskal–Wallis tests identified significant differences in alpha diversity in leaves between sites ($p < 0.001$, Figure 1D). Here we report the results of Bonferroni corrected *post hoc* Dunn tests which were used to assess which pair-wise site comparisons were driving observed differences. Pair-wise comparisons that are significantly different are bolded ($p < 0.05$) and shaded gray. Full site names can be found in Table 2.

	AK	WA	BB	SD	QU	MA	NC	NN	SW	UK	PO	FR	CR	JS	JN
AI	**0.0125**	0.8851	0.6186	0.1711	0.5596	0.8169	0.1786	0.9647	0.3550	0.3014	0.8019	0.1509	0.9719	0.8690	0.7180
AK		**0.0193**	0.1126	**0.0001**	0.0691	**0.0032**	**0.0001**	0.0953	0.5034	0.4079	**0.0119**	**0.0001**	**0.0341**	**0.0228**	**0.0022**
WA			0.7243	0.1150	0.6876	0.6861	0.1085	0.9652	0.4135	0.3607	0.6878	0.1193	0.8695	0.9894	0.6012
BB				0.0580	0.9947	0.4138	0.0572	0.7397	0.6989	0.6543	0.4171	0.0548	0.6461	0.7446	0.3556
SD					**0.0325**	0.2985	0.9681	0.3202	**0.0372**	**0.0196**	0.3860	0.8092	0.3144	0.1105	0.3627
QU						0.3686	**0.0360**	0.7215	0.6825	0.6218	0.3849	**0.0362**	0.6100	0.7146	0.3098
MA							0.3154	0.8466	0.2654	0.1811	0.9596	0.2573	0.8984	0.6931	0.9077
NC								0.3280	**0.0374**	**0.0220**	0.4170	0.7366	0.3145	0.1090	0.3851
NN									0.4677	0.4212	0.8139	0.2711	0.9664	0.9716	0.7595
SW										0.9984	0.2865	**0.0361**	0.3706	0.4154	0.2171
UK											0.2209	**0.0204**	0.3288	0.3675	0.1413
PO												0.3189	0.8707	0.6962	0.9666
FR													0.2558	0.1157	0.3051
CR														0.8657	0.8166
JS															0.5963

Table S5. Pair-wise *post hoc* Dunn tests of sediment Shannon diversity in the ITS2 region dataset. Kruskal–Wallis tests identified significant differences in alpha diversity in leaves between sites ($p < 0.001$, Figure 1B). Here we report the results of Bonferroni corrected *post hoc* Dunn tests which were used to assess which pair-wise site comparisons were driving observed differences. Pair-wise comparisons that are significantly different are bolded ($p < 0.05$) and shaded gray. Full site names can be found in Table 2.

	AK	WA	BB	SD	QU	MA	NC	NN	SW	UK	PO	FR	CR	JS	JN
AI	0.7310	0.8553	0.4187	0.2857	0.6586	0.2975	0.9503	0.8645	**0.0095**	0.6602	0.5377	0.7709	0.4207	**0.0017**	0.8048
AK		0.8610	0.6585	0.5436	0.9041	0.4289	0.7745	0.6570	**0.0031**	0.4170	0.7637	0.9696	0.2210	**0.0007**	0.6018
WA			0.5491	0.4105	0.7825	0.3604	0.8920	0.7706	**0.0068**	0.5507	0.6638	0.8494	0.3236	**0.0017**	0.7088
BB				0.9064	0.7057	0.6690	0.4716	0.3448	**0.0014**	0.1835	0.8495	0.7029	0.0724	**0.0003**	0.3251
SD					0.5997	0.7152	0.3279	0.2223	**0.0002**	0.0703	0.7721	0.6394	**0.0212**	**0.0000**	0.2031
QU						0.4727	0.7126	0.5783	**0.0016**	0.3299	0.8114	0.9471	0.1596	**0.0003**	0.5332
MA							0.3261	0.2486	**0.0051**	0.1542	0.5951	0.4815	0.0766	**0.0018**	0.2247
NC								0.8539	**0.0110**	0.6558	0.5878	0.7888	0.4239	**0.0022**	0.7774
NN									**0.0205**	0.7682	0.4413	0.6845	0.5400	**0.0039**	0.9068
SW										0.0562	**0.0017**	**0.0126**	0.2185	0.6854	**0.0497**
UK											0.2428	0.4970	0.7113	**0.0100**	0.8477
PO												0.8071	0.1097	**0.0003**	0.4160
FR													0.3237	**0.0032**	0.6510
CR														0.0616	0.6356
JS															**0.0104**

Table S6. Pair-wise *post hoc* Dunn tests of sediment Shannon diversity in the 18S rRNA gene dataset. Kruskal–Wallis tests identified significant differences in alpha diversity in leaves between sites ($p < 0.001$, Figure 1D). Here we report the results of Bonferroni corrected *post hoc* Dunn tests which were used to assess which pair-wise site comparisons were driving observed differences. Pair-wise comparisons that are significantly different are bolded ($p < 0.05$) and shaded gray. Full site names can be found in Table 2.

	AK	WA	BB	SD	QU	MA	NC	NN	SW	UK	PO	FR	CR	JS	JN
AI	0.8675	0.1621	0.6253	0.1261	0.9131	0.1724	0.9605	0.7093	**0.0168**	0.8820	0.2997	0.1693	0.9939	0.0814	0.1818
AK		0.1016	0.4976	0.0639	0.6998	0.1294	0.9109	0.8884	**0.0171**	0.6674	0.1615	0.1262	0.8274	0.1012	0.1013
WA			0.5456	1.0000	0.1811	0.9454	0.1627	0.0798	**0.0009**	0.1747	0.5814	0.9371	0.1501	**0.0032**	0.7083
BB				0.5274	0.7069	0.5418	0.6096	0.3960	**0.0207**	0.7011	0.8904	0.5346	0.6246	0.0652	0.7564
SD					0.1592	0.9584	0.1324	0.0533	**0.0002**	0.1471	0.5407	0.9499	0.1067	**0.0010**	0.6650
QU						0.2027	0.8833	0.5949	**0.0088**	0.9867	0.4190	0.1995	0.9061	**0.0497**	0.2516
MA							0.1778	0.1128	**0.0034**	0.2021	0.5659	1.0000	0.1768	**0.0107**	0.6769
NC								0.7908	**0.0424**	0.8617	0.3023	0.1744	0.9426	0.1292	0.1900
NN									0.0612	0.5359	0.1273	0.1095	0.6580	0.1810	0.0806
SW										**0.0037**	**0.0002**	**0.0031**	**0.0074**	0.6167	**0.0001**
UK											0.3974	0.1988	0.8780	0.2247	**0.0245**
PO												0.5589	0.2637	**0.0020**	0.8684
FR													0.1733	0.0513	0.1730
CR														**0.0099**	0.6693
JS															**0.0007**

Table S7. Evidence of distance-decay from Mantel test results. After first subsetting each dataset (ITS2 region, 18S rRNA gene) by ocean (Pacfic, Atlantic) and sample type (leaf, root, sediment), we identified distance-decay patterns by testing for correlations between the community distance (Bray-Curtis, Hellinger) and the geographic distance between samples. Here we report the results of each Mantel test. We also report the results of each Mantel test when samples from the same site are excluded (e.g. all samples at a geographic distance of zero) as indicated in the table by an asterisk.

Ocean	Amplicon dataset	Sample type	Beta diversity metric	Mantel statistic (*r*)	*p*-value	Mantel statistic (*r*)*	*p*-value*
**Pacific**	ITS2 region	Leaf	Bray-Curtis	0.197	**0.0001**	0.0699	**0.0291**
**Pacific**	ITS2 region	Leaf	Hellinger	0.1767	**0.0001**	0.0671	**0.0376**
**Pacific**	18S rRNA gene	Leaf	Bray-Curtis	0.4589	**0.0001**	0.1438	**0.0034**
**Pacific**	18S rRNA gene	Leaf	Hellinger	0.4202	**0.0001**	0.1580	**0.0037**
**Pacific**	ITS2 region	Root	Bray-Curtis	0.2195	**0.0001**	0.0529	0.0785
**Pacific**	ITS2 region	Root	Hellinger	0.1869	**0.0001**	0.0309	0.2007
**Pacific**	18S rRNA gene	Root	Bray-Curtis	0.0696	**0.03**	-0.1035	0.9896
**Pacific**	18S rRNA gene	Root	Hellinger	0.09	**0.0094**	-0.0638	0.9129
**Pacific**	ITS2 region	Sediment	Bray-Curtis	0.1417	**0.0002**	-0.0188	0.7012
**Pacific**	ITS2 region	Sediment	Hellinger	-0.03682	0.8238	-0.1093	0.9788

**Pacific**	18S rRNA gene	Sediment	Bray-Curtis	0.3725	**0.0001**	0.1122	0.9983
**Pacific**	18S rRNA gene	Sediment	Hellinger	0.2352	**0.0001**	-0.0675	0.8675
**Atlantic**	ITS2 region	Leaf	Bray-Curtis	0.1139	**0.0002**	-0.044	0.9544
**Atlantic**	ITS2 region	Leaf	Hellinger	0.1057	**0.0001**	-0.044	0.9527
**Atlantic**	18S rRNA gene	Leaf	Bray-Curtis	0.1639	**0.0001**	0.0418	0.1351
**Atlantic**	18S rRNA gene	Leaf	Hellinger	0.1793	**0.0001**	0.0495	0.0967
**Atlantic**	ITS2 region	Root	Bray-Curtis	0.1655	**0.0001**	0.0143	0.3019
**Atlantic**	ITS2 region	Root	Hellinger	0.1646	**0.0001**	0.0075	0.3819
**Atlantic**	18S rRNA gene	Root	Bray-Curtis	0.4632	**0.0001**	0.3441	**0.0001**
**Atlantic**	18S rRNA gene	Root	Hellinger	0.4216	**0.0001**	0.3101	**0.0001**
**Atlantic**	ITS2 region	Sediment	Bray-Curtis	0.2713	**0.0001**	0.171	**0.0001**
**Atlantic**	ITS2 region	Sediment	Hellinger	0.2094	**0.0001**	0.1283	**0.0034**
**Atlantic**	18S rRNA gene	Sediment	Bray-Curtis	0.2592	**0.0001**	0.0781	**0.0486**
**Atlantic**	18S rRNA gene	Sediment	Hellinger	0.2072	**0.0001**	0.0753	0.0802

Table S8. Predicted core ITS2 region ASVs. ASVs were ranked by their abundance-occupancy distribution and then predicted to be in the core based on a final percent increase of equal or greater than 10% to Bray-Curtis dissimilarity. The Sloan neutral model was then applied to the abundance-occupancy distributions to identify ASVs that deviate such that ASVs above the neutral model are predicted to be selected for by the environment (e.g. by the host plant, *Z. marina*), and those below the model are predicted to be selected-against or dispersal-limited. Here we report the core ASVs for the *Z. marina* leaf, root and sediment mycobiomes, whether these ASVs deviate from the neutral model (above, below, none) and the taxonomy of each ASV.

ASV	Core prediction	Neutral model deviation	Taxonomy
**ITS_SV45**	leaf, root, sediment	above, above, above	*Cladosporium* sp.
**ITS_SV52**	leaf, root, sediment	above, above, none	*Mycosphaerella tassiana*
**ITS_SV154**	leaf, root, sediment	none, none, above	*Alternaria alternata*
**ITS_SV260**	leaf, root	none, none	*Saccharomyces paradoxus*
**ITS_SV362**	leaf	below	*Lobulomyces* sp.
**ITS_SV381**	leaf	above	*Aureobasidium pullulans*
**ITS_SV389**	leaf	none	*Lecanora populicola*
**ITS_SV426**	leaf, sediment	none, none	*Saccharomyces* sp.
**ITS_SV779**	leaf, root, sediment	above, above, none	*Malassezia restricta*

**ITS_SV807**	leaf	none	*Meliniomyces* sp.
**ITS_SV1714**	leaf	above	Unclassified Capnodiales sp.
**ITS_SV2181**	leaf	none	Unclassified Capnodiales sp.
**ITS_SV2377**	leaf	none	Unclassified Capnodiales sp.
**ITS_SV5566**	leaf	none	*Malassezia globosa*
**ITS_SV60**	root	below	Unclassified Sordariomycetes sp.
**ITS_SV101**	root	none	Unclassified Didymellaceae sp.
**ITS_SV125**	root	none	Unclassified Ascomycota sp.
**ITS_SV169**	root	none	Unclassified Didymellaceae sp.
**ITS_SV234**	root	none	Unclassified Sordariomycetes sp.
**ITS_SV497**	root	below	Unclassified Sordariomycetes sp.
**ITS_SV540**	root	none	*Phaeotheca salicorniae*
**ITS_SV590**	root	above	*Cladosporium halotolerans*
**ITS_SV766**	root	none	*Phaeotheca salicorniae*
**ITS_SV1045**	root	above	*Hortaea werneckii*
**ITS_SV31**	sediment	above	*Cladosporium* sp.
**ITS_SV144**	sediment	none	*Cystobasidium pinicola*

**ITS_SV210**	sediment	above	*Didymella glomerata*
**ITS_SV404**	sediment	below	Unclassified Pleosporaceae sp.
**ITS_SV630**	sediment	above	*Paraphaeosphaeria angularis*
**ITS_SV679**	sediment	above	*Pseudeurotium bakeri*
**ITS_SV880**	sediment	below	Unclassified fungal sp.
**ITS_SV950**	sediment	above	*Pyrenochaetopsis leptospora*
**ITS_SV952**	sediment	none	Unclassified Sclerotiniaceae sp.
**ITS_SV962**	sediment	above	*Paraconiothyrium cyclothyrioides*
**ITS_SV1255**	sediment	above	*Wickerhamomyces anomalus*
**ITS_SV1468**	sediment	above	*Paraconiothyrium brasiliense*
**ITS_SV1538**	sediment	above	*Pyrenochaetopsis leptospora*
**ITS_SV1745**	sediment	above	*Trichoderma* sp.
**ITS_SV1928**	sediment	above	Unclassified Hyaloscyphaceae sp.
**ITS_SV2045**	sediment	above	*Saitozyma podzolica*
**ITS_SV2047**	sediment	above	*Psathyrella maculata*
**ITS_SV2164**	sediment	above	Unclassified Saccharomycetales sp.
**ITS_SV2245**	sediment	none	Unclassified Rhizophydiales sp.

**ITS_SV2430**	sediment	above	*Ganoderma* sp.
**ITS_SV2439**	sediment	above	*Penicillium* sp.
**ITS_SV2488**	sediment	none	*Trichoderma* sp.
**ITS_SV2529**	sediment	above	*Lycoperdon nigrescens*
**ITS_SV2601**	sediment	above	*Paraphaeosphaeria* sp.
**ITS_SV2610**	sediment	above	*Paraconiothyrium archidendri*
**ITS_SV2625**	sediment	above	*Paraconiothyrium* sp.
**ITS_SV2685**	sediment	above	Unclassified Pleosporales sp.
**ITS_SV2716**	sediment	none	*Lycoperdon pyriforme*
**ITS_SV2876**	sediment	none	Unclassified Aphelidiomycota sp.
**ITS_SV2885**	sediment	above	*Lachancea mirantina*
**ITS_SV3107**	sediment	above	Unclassified Teichosporaceae sp.
**ITS_SV3162**	sediment	above	*Didymocyrtis cladoniicola*
**ITS_SV3286**	sediment	above	*Penicillium* sp.
**ITS_SV3401**	sediment	above	*Coprinellus* sp.
**ITS_SV3483**	sediment	above	*Trichoderma* sp.
**ITS_SV3636**	sediment	above	*Fomitiporella* sp.

**ITS_SV3851**	sediment	above	Unclassified Microbotryales sp.
**ITS_SV4118**	sediment	none	*Hypholoma fasciculare*
**ITS_SV4193**	sediment	above	*Myrothecium* sp.
**ITS_SV5121**	sediment	above	*Trichoderma* sp.
**ITS_SV5439**	sediment	none	*Pyrenochaetopsis tabarestanensis*
**ITS_SV5656**	sediment	none	*Paraphaeosphaeria angularis*
**ITS_SV5741**	sediment	above	*Pyrenochaetopsis leptospora*
**ITS_SV5941**	sediment	none	*Cortinarius bivelus*
**ITS_SV6868**	sediment	above	*Endoxyla* sp.
**ITS_SV7233**	sediment	above	*Lophiostoma* sp.
**ITS_SV7652**	sediment	none	Unclassified Hypocreales sp.
**ITS_SV8621**	sediment	none	*Betamyces* sp.
**ITS_SV11477**	sediment	above	*Coprinellus micaceus*
**ITS_SV11928**	sediment	none	Chytridiomycetes sp.
**ITS_SV12885**	sediment	above	*Phialocephala humicola*
**ITS_SV13529**	sediment	above	*Phaeosphaeriaceae* sp.
**ITS_SV16671**	sediment	none	*Hypholoma capnoides*

**ITS_SV21975**	sediment	none	Unclassified Dictyosporiaceae sp.
**ITS_SV50231**	sediment	none	Unclassified Gigasporales sp.

Table S9. Predicted core 18S rRNA gene ASVs. ASVs were ranked by their abundance-occupancy distribution and then predicted to be in the core based on a final percent increase of equal or greater than 10% to Bray-Curtis dissimilarity.The Sloan neutral model was then applied to the abundance-occupancy distributions to identify ASVs that deviate such that ASVs above the neutral model are predicted to be selected for by the environment (e.g. by the host plant, *Z. marina*), and those below the model are predicted to be selected-against or dispersal-limited. Here we report the core 18S rRNA gene ASVs for the *Z. marina* leaf, root and sediment mycobiomes, whether these ASVs deviate from the neutral model (above, below, none) and the taxonomy of each ASV.

ASV	Core prediction	Neutral model deviation	Taxonomy
**18S_SV756**	leaf, root	none, none	Unclassified Chytridiomycetes sp.
**18S_SV928**	leaf, root	above, above	*Saccharomyces* sp.
**18S_SV968**	leaf	none	Unclassified Lobulomycetaceae sp.
**18S_SV1004**	leaf	none	*Saccharomyces* sp.
**18S_SV1553**	leaf	none	Unclassified Gromochytriaceae sp.
**18S_SV1567**	leaf, root	none, none	Unclassified Sordariomycetes sp.
**18S_SV1734**	leaf, root	none, none	Unclassified Rhizophydiales sp.
**18S_SV1977**	leaf, root	none, none	*Chytridium* sp.

**18S_SV3823**	leaf	none	*Cryptococcus* sp.
**18S_SV970**	root	none	Unclassified Sordariomycetes sp.
**18S_SV2546**	root	none	Unclassified Chytridiomycota sp.
**18S_SV3446**	root	none	Unclassified Aphelidea sp.
**18S_SV4479**	root	below	Unclassified Sordariomycetes sp.
**18S_SV5426**	root	above	Unclassified Chytridiomycota sp.
**18S_SV8081**	root	none	Unclassified Lobulomycetaceae sp.
**18S_SV10835**	root	none	*Chytridium* sp.
**18S_SV18125**	root	none	Unclassified Lobulomycetaceae sp.
**18S_SV27158**	root	none	Unclassified Chytridiomycetes sp.
**18S_SV897**	sediment	none	Unclassified Chytridiomycetes sp.
**18S_SV1751**	sediment	none	Unclassified Rhizophydiales sp.
**18S_SV2055**	sediment	above	*Trichoderma* sp.
**18S_SV2127**	sediment	none	Unclassified Spizellomycetales sp.
**18S_SV2138**	sediment	none	Unclassified Chytridiomycetes sp.
**18S_SV3230**	sediment	below	*Metschnikowia* sp.
**18S_SV5260**	sediment	none	*Metschnikowia* sp.

**18S_SV8806**	sediment	none	*Blastobotrys* sp.
**18S_SV14838**	sediment	below	Unclassified Boletales sp.
**18S_SV18689**	sediment	none	Unclassified Ascomycota sp.
**18S_SV20875**	sediment	none	Unclassified Rhizophydiales sp.
**18S_SV23021**	sediment	none	Unclassified Agaricales sp.
**18S_SV23280**	sediment	none	*Rhizophydium* sp.
